# Lower Carbon Footprint Concrete Using Recycled Carbon Fiber for Targeted Strength and Insulation

**DOI:** 10.3390/ma16155451

**Published:** 2023-08-03

**Authors:** Andrew Patchen, Stephen Young, Logan Goodbred, Stephen Puplampu, Vivek Chawla, Dayakar Penumadu

**Affiliations:** Tickle College of Engineering, The University of Tennessee, Knoxville, TN 37996, USA

**Keywords:** fiber-reinforced concrete, recycled carbon fiber, mechanical properties, X-ray tomography

## Abstract

The production of concrete leads to substantial carbon emissions (~8%) and includes reinforcing steel which is prone to corrosion and durability issues. Carbon-fiber-reinforced concrete is attractive for structural applications due to its light weight, high modulus, high strength, low density, and resistance to environmental degradation. Recycled/repurposed carbon fiber (rCF) is a promising alternative to traditional steel-fiber reinforcement for manufacturing lightweight and high-strength concrete. Additionally, rCF offers a sustainable, economical, and less energy-intensive solution for infrastructure applications. In this paper, structure–process–property relationships between the rheology of mix design, carbon fiber reinforcement type, thermal conductivity, and microstructural properties are investigated targeting strength and lighter weight using three types of concretes, namely, high-strength concrete, structural lightweight concrete, and ultra-lightweight concrete. The concrete mix designs were evaluated non-destructively using high-resolution X-ray computed tomography to investigate the microstructure of the voids and spatially correlate the porosity with the thermal conductivity properties and mechanical performance. Reinforced concrete structures with steel often suffer from durability issues due to corrosion. This paper presents advancements towards realizing concrete structures without steel reinforcement by providing required compression, adequate tension, flexural, and shear properties from recycled/repurposed carbon fibers and substantially reducing the carbon footprint for thermal and/or structural applications.

## 1. Introduction

### 1.1. Overview: Fiber-Reinforced Ultra-Lightweight, Structural Lightweight, and High-Strength Concrete

Concrete is a versatile material utilized in civil infrastructure for applications ranging from simple architectural features to critical structural elements in bridges and buildings [1,2,3,4]. The concrete industry produces 4.4 billion metric tons of cement worldwide, a figure which has been steadily increasing year to year [5]. Due to the many different applications of concrete, the mix designs must be tailored to meet numerous different specifications and requirements. Energy-efficient buildings are under environmental requirements to reduce CO_2_ at lower cost; thus, there is a need to develop lightweight and high-strength concrete to improve or develop construction materials [6,7,8,9,10,11]. Thermal conductivity is a key property that affects the insulation characteristics of buildings. Reduction in energy consumption coupled with sustainable design can decrease thermal losses in buildings [12]. Among the primary factors affecting the thermal conductivity of concrete are its porosity and density [13,14]. Therefore, lightweight aggregate concrete (LWAC) is attractive for buildings and concrete floor slab applications as it has lower density and better thermal insulation than normal concrete [7,15,16,17]. Lightweight aggregates are derived from materials such as perlite, vermiculite, pumice, expanded-clay mix, sintered fly ash, coconut shell, and expanded shale, to name a few, which are incorporated in concrete to entrain air in lightweight concrete designs [7,18,19]. A commonly used structural application for lightweight concrete is decking for bridges and building floors; here, concrete mixes that are both strong and lightweight are utilized in the form of structural lightweight concrete that can reduce the overall weight of the structure while still providing adequate strength, thus increasing the building efficiency [20,21]. Furthermore, for architectural details and esthetic pieces, where the strength of the concrete is not critical, the use of ultra-lightweight concrete can make installation easier and reduce the load on the structure [22,23,24]. Additionally, due to its air voids ultra-lightweight concrete can provide superior thermal insultation properties to traditional concrete [25].

Aggregates such as manufactured sand (M sand), consisting of crushed sand, have been investigated as a sustainable aggregate due to their lower cost and have been used in high-strength concrete structural applications, including mix designs with lightweight aggregates [26,27,28,29]. If effective concrete mix designs for different applications are to be developed, a comprehensive understanding of concrete’s workability, rheological and mechanical properties, thermal characteristics, and many other properties is essential [30]. For all the differing concrete mixes and applications, concrete has one major drawback: its tensile strength is typically much lower than its compressive strength. Therefore, a typical reinforced concrete design does not consider the tensile strength of the concrete. To carry tensile loads, a steel rebar is embedded into the concrete, and the tensile stress is transferred from the concrete to the embedded rebar [2]. However, a rebar is both expensive and time-consuming to install and makes up about 18% of the cost of concrete construction [31]. Additionally, a rebar can corrode, leading to reduced strength and rust jacking that damages the surrounding concrete [32,33,34]. This reduced strength from corrosion of the rebar-reinforced concrete is largely due to the loss of adhesion between the rebar and the concrete [35,36].

Fiber-reinforced cementitious and concrete have been investigated to explore sustainable solutions for structural applications [37,38,39,40]. Fiber-reinforced concrete (FRC) has been incorporated into LWAC and normal concrete and can potentially improve the tensile strength of concrete [26,27,28,29,41,42]. Henceforth, as defined for this study, FRC consists of randomly oriented short fibers mixed into the concrete to serve as a form of tensile reinforcement [30,43]. FRC improves the tensile properties of the concrete by transferring the tensile stresses within the concrete to the fibers through an interfacial bond between the fiber and surrounding concrete matrix [44,45,46,47]. The fibers possess significantly higher tensile strength than neat concrete, allowing them to carry higher tensile force and resulting in increased tensile strength and durability for FRC [2,44,45,46,47]. Several fiber types have been studied, including polypropylene, glass, basalt, and natural fibers, and incorporated into concrete to tailor the performance of different concrete mixes for varied applications [30,48,49,50,51]. Traditionally, steel fibers have been the fiber type most commonly used in FRC structural applications due to their enhancement of mechanical properties and relatively low cost [48,52,53]. Typically, high-strength concrete is used in these applications, as it has a strength greater than 50 MPa [54,55,56,57,58,59,60,61,62,63,64]. P.S. Song et al. evaluated the use of steel fibers in high-performance concrete and found that the impact resistance was greatly increased with the addition of steel fibers [44]. P. Balaguru and A. Foden investigated structural lightweight concrete reinforced with steel fibers and found that steel fibers significantly increased split tensile strength and flexural strength and moderately increased compressive strength [65]. J. Wang et al. observed that the addition of steel fibers increased the density, as well as the compressive and flexural strength, of the concrete [66]. A potential alternative to steel fiber is carbon fiber, which is promising for FRC applications due to its high modulus, strength, and chemical inertness [67,68]. 

### 1.2. Carbon Fiber and Recycled Carbon Fiber Overview

As carbon fibers have higher tensile strength and modulus than steel fibers, they can carry higher tensile forces in concrete [67,69]. A. B. Kizikanat evaluated high-strength concrete with carbon-fiber reinforcement and found that the flexural and split tensile strength significantly increased with the addition of carbon fiber, and the compressive strength increased slightly [70]. Few studies have investigated carbon fiber as a reinforcement for ultra-lightweight concrete or examined how it affects the thermal properties of the concrete. B. Chen and J. Liu explored lightweight concrete reinforced with polypropylene fiber, carbon fiber, steel fiber, and hybrid combinations of the different fibers in compression and split tension [71]. They found that the compressive strength and split tensile strength increased with the addition of carbon fiber, and the combination of steel and carbon fiber further increased the compressive strength [71]. Additionally, the steel fiber and carbon fiber hybrid mix had the highest toughness index [71]. Thus, carbon fiber could be an ideal replacement for steel fiber in concrete; however, carbon fibers can be more costly than steel fibers, potentially making them much less economical [72,73,74]. One possible way to reduce the cost is to utilize recycled carbon fiber (rCF) [75,76,77].

Carbon fiber has not yet realized its full potential commercial utilization cost for market entry, which has limited its use outside of high-performance applications such as aerospace and high-end performance vehicles. The conversion of a precursor, such as polyacrylonitrile (PAN), to carbon fiber is energy- and cost-intensive, and a significant amount of waste carbon fiber is disposed into landfills when end-of-the-lifecycle-part stage is reached [78,79,80,81,82,83]. Waste carbon-fiber-reinforced polymers (CFRP) from the automotive and aerospace industries can be recycled for use in concrete, helping to prevent the accumulation of CFRP in landfills [77,84,85]. The CFRP waste can be recycled into fibers through three processes: thermal, chemical, and mechanical [77]. However, the recycling of carbon fiber is difficult and can lead to fiber damage, fiber length variation, fiber diameter change, contamination, and loss of strength, as well as potentially producing toxic fumes [76,77,86,87,88,89,90,91,92,93,94,95,96,97,98,99].

Studies have shown the use of recycled CFRP in concrete. Ogi et al. investigated the flexural and compressive strength and fracture behavior of concrete reinforced with recycled CFRP pieces [100]. Additionally, Mastali et al. showed that recycled CFRP improved the compressive strength, flexural strength, and impact resistance of self-compacting concrete [101,102]. However, although the fiber length and volume fraction increased the impact resistance, the workability of the concrete was reduced, leading to increased defects and voids [102]. 

The surface of the carbon fiber can play an important role in the fiber-to-cement bond. Fibers with a rough surface have a stronger attachment to the cement than those with a smoother surface [103,104,105]. It is therefore important to understand the microstructure of the carbon fibers. Akbar et al. studied the surface of milled rCF by using scanning electron microscopy (SEM) combined with energy X-ray dispersive spectroscopy (EDS), X-ray diffraction (XRD), thermogravimetric analysis (TGA), and Fourier transform infrared spectroscopy (FTIR) [84]. Through XRD, TGA, and FTIR, Akbar et al. found that the defects on the surface of the milled rCF created locations for nucleation for Portland cement [84]. This improved bonding, combined with the uniform dispersion of the fibers, helps to improve the compressive and flexural strength of cement paste compared to neat cement paste [84]. Wide angle X-ray scattering (WAXS) is an invaluable tool to characterize amorphous and crystalline regions within polymer materials and fibrous materials such as carbon fiber, whose crystalline parameters have been correlated to single-fiber mechanical properties [106,107,108]. 

### 1.3. Micro X-ray Computed (µ-XCT) and Thermal Conductivity of Concrete Mix Designs

X-ray computed tomography (XCT) is an effective technique to evaluate complex geometry, including lightweight concrete, M sand, and high-performance concrete, by detecting microstructural features of interest such as air voids, fiber, and concrete phases [109,110,111,112,113]. Furthermore, the addition of fibers can affect the thermal conductivity of the concrete due to the fibers having a higher thermal conductivity than the concrete matrix [114,115]. K. Liu et al. investigated how the addition of carbon and steel fiber affects the thermal conductivity of normal-weight concrete. They found that as the volume fraction of steel fiber increased, so did the thermal conductivity. For the carbon-fiber-based mixes, they found a similar trend, that is, the thermal conductivity increased with increased fiber content; however, if the mix did not have adequate workability, the addition and pore dispersion of the fibers could lead to it having decreased thermal conductivity [114]. 

### 1.4. Significance of This Study

The main knowledge gaps in the literature involve the consideration of recycled carbon fibers which dramatically reduce the carbon footprint by capturing the high embodied energy needed to manufacture virgin carbon fibers and identifying them for augmenting tensile properties to minimize or eliminate reinforcing steel rebars. High performance concrete is being considered currently by many state and federal transportation projects and this paper provides an alternative to replace steel fibers with much more durable and environmentally friendly recycled carbon fibers. In addition to structurally demanding applications, concrete is utilized extensively for other applications that require improved thermal insulation. This paper addresses these critical knowledge gaps and also overcomes the limited studies that exist on the structure–process–property relationship of carbon fibers utilized in ultra-lightweight, structural lightweight, and high-strength concrete. Thus, there is a need for a comprehensive study of the microstructure of the interfacial bonds between the fiber, fiber sizing, and surrounding concrete matrix, including microstructural features such as voids. In this study, a multiscale analysis experimental program was developed to evaluate the influence of rCF-reinforced concrete on three concrete mixes made from three aggregate types compared to a commercially available steel fiber reinforcement. The three concrete mix designs incorporating rCF were high-strength, structural-light, and ultra-light concrete. The rCF-reinforced concrete designs were evaluated for their mechanical properties, such as compression, tensile, and flexural strength. The crystal lattice parameters of the rCF were calculated using a WAXS technique to provide insight into the structural–mechanical performance of the fibers. The surface morphology of the fibers was examined using SEM, and the elemental chemical composition of the fibers was observed using energy dispersive X-ray spectroscopy (EDS). Furthermore, high-resolution micro X-ray computed tomography (µ-XCT) was utilized to analyze the pore geometry content spatially in 3D to investigate porosity structure in relation to each concrete mix design’s mechanical performance. The thermal conductivity of the tensile samples was measured to investigate the relationship between the porosity obtained from µ-XCT for each concrete mix design. The thermal conductivity results were compared with reconstructed 3D volumes obtained through µ-XCT to investigate how the voids and fiber distribution affect the thermal conductivity of the different concrete mixes. The surface of mechanically failed tensile samples was imaged using SEM to observe the failure mechanism of the fibers and concrete. This study highlights novel insights on the effects of rCF when incorporated into various concrete mix designs for lightweight and high-strength concrete structural applications, including the microstructure and porosity effects of the final concrete mix, thermal insulation effects, and the fiber reinforcement failure mechanisms of the concrete mix.

## 2. Materials and Methods

### 2.1. Experimental Program for the Concrete Mix Designs

A multiscale analytical experimental program was used in this study to characterize the concrete mix design and was divided into three stages. The first stage involved characterizing the fibers for their structural properties. The microstructure of the fibers was examined using SEM, and the chemical elemental composition of each fiber type was identified using EDS. Additionally, the structure of the recycled carbon fibers was evaluated for their crystalline structure using a WAXs technique. In the second stage, the concrete mix design was evaluated non-destructively using µ-XCT to investigate the void volume content spatially and the effects of void formation from incorporating fibers in the concrete mixes coupled with the resulting reinforcement mechanisms between the host concrete matrix and surrounding the fibers [111]. The third stage consisted of evaluating the concrete mix designs for compressive, tensile, and flexural strength properties. The failed tensile briquette samples were then evaluated for their thermal conductivity properties. The mechanical properties were correlated with the porosity and thermal conductivity properties of the concrete mixes. 

### 2.2. Fiber Properties

**Figure 1:**
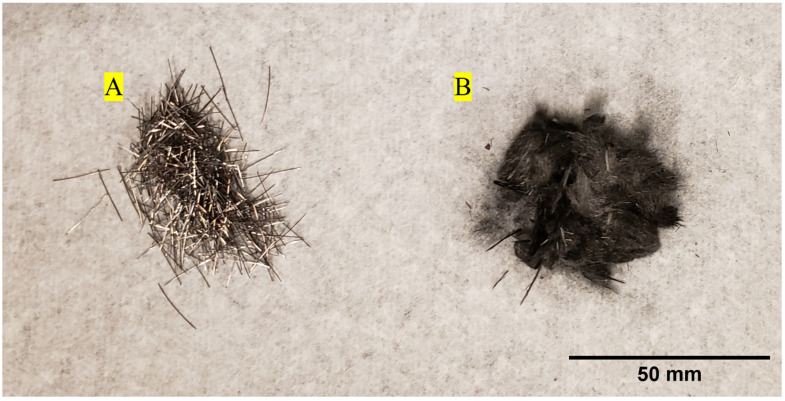
Fiber types (**A**): steel fiber and (**B**): recycled carbon fiber mixed into the concrete mix designs.

This study involved the use of rCF and steel fiber as reinforcement for concrete. The rCF used for this study was supplied by Carbon Fiber Recycling LLC (Tazewell, TN, USA) and was used as received. The steel fibers were manufactured in accordance with ASTM A820 type 1 with a 13 mm length [116]. Figure 1 shows the fiber types used in this work, namely steel fiber and rCF. The physical properties of the fibers, including density, length, and diameter, are summarized in Table 1, and detailed characterizations are given in a previous study [111]. Briefly, the rCF fiber length and diameter were measured using a high-resolution digital microscope (Keyence, VHX 7000 series, Itasca, IL, USA); 70 fibers were measured for length, and 38 measurements were taken for diameter. Additionally, the densities for the rCF were measured using a gas pycnometer (Micromeritics, AccuPyc II 1340, Norcross, GA, USA). The fiber-sizing content for rCF was measured as 0.99% based on a TGA conducted in a previous study [111].

### 2.3. Scanning Electron Microscopy and Energy Dispersive X-ray Spectroscopy of Fibers

The surface morphology of the fibers was examined using SEM, specifically, a ThermoFisher Scientific Apreo S SEM instrument (ThermoFisher Scientific, Waltham, MA, USA) with an accelerating voltage of 20 kV. Additionally, the chemical elemental composition was observed using EDS. The fibers were Au sputter coated using a sputter coater (SPI-Module Sputter Coater, West Chester, PA, USA) for 40 s. An environmental SEM (Zeiss SEM EVO^®^ MA15, Carl Zeiss, Oberkochen, Germany) was used to examine the surface topography of the tensile briquette samples after mechanical failure with an accelerating voltage of 20 kV using variable pressure [111].

### 2.4. Wide Angle X-ray Scattering of Carbon Fiber

**Figure 2:**
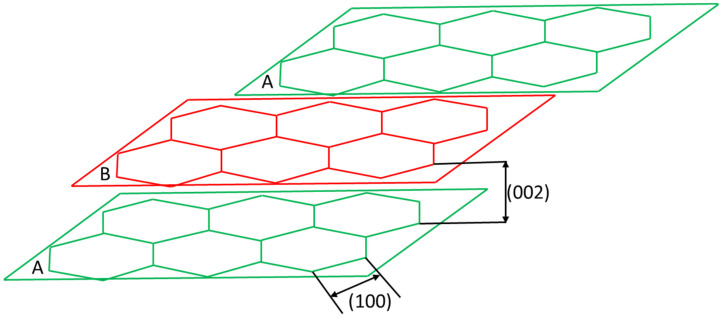
Illustration of graphitic lattice structure showing the d-spacing for 002 and 100. The planes are designated A and B to illustrate the AB stacking sequence of the graphene [106].

WAXS was performed to examine the crystalline microstructure of the fibers for two different batches of rCF and a reference commercial carbon fiber (T700). The scanning was performed on a Xenocs GeniX 3D microfocus instrument equipped with a Cu K-alpha 1.54 Å X-ray source with a voltage of 50 kV and a current of 0.60 mA over a 2θ angular range of approximately 0 to 55 degrees. The d-spacings, crystal size (L_a_), and stacking height (L_c_) were calculated using the following two relationships based on Bragg’s law (Equation (1)) and the Debye–Scherrer equation (Equation (2)):

(1) $$d_{i}=\frac{\lambda }{2sin\theta }$$

(2) $$L_{n}=\frac{K\lambda }{\beta cos\theta }$$

where *λ* is the wavelength, *θ* is the Bragg angle in radians, and *d_i_* is the interlayer distance between the crystalline planes determined from the peak position of the diffracting planes 002 and 100 as illustrated in Figure 2. Additionally, the crystalline parameters L_n_ (L_c_ or L_a_) are based on Bragg’s law, and *K* is Scherrer shape factor constant corresponding to a value of 0.9 for L_c_ and 1.8 for L_a_. *β* is the FWHM corresponding to 002 and 100 reflections or peaks corrected for instrument broadening [106,118,119]. The T700, a standard modulus carbon fiber used in the industry, was used as a reference carbon fiber for comparison with the rCF samples [120].

### 2.5. High-Strength and Lightweight Concrete Mix Designs

Three types of concretes were utilized for the concrete mix designs in this study. The first was a high-strength concrete mix design for structural applications. The second was structural-light concrete typically used for floor slabs and other applications where both the strength and density of the concrete are essential properties. The third was ultra-light concrete for increased thermal insulation and lower density than the two previously mentioned types. To investigate the effect of rCF, three mixes were reinforced with rCF along with two control mixes, namely a mix reinforced with steel fibers and a neat concrete mix containing no fiber reinforcement. Henceforth, the three types of concrete in this study are identified as high-strength, structural-light, and ultra-light. 

Table 2 summarizes the mix designs used in this study, presented in kilograms of material per cubic meter of the final concrete mix. The mix design for each of the three base mixes utilized a Type I all-purpose Portland cement and silica fume (MasterLife SF 100, Cleveland, OH, USA) as the cementitious materials and a 0.3 water-to-cementitious materials ratio. The ratio of cementitious materials was 90% Portland cement and 10% silica fume by volume. The Portland cement and silica fume react with water and bind the concrete mix constituents together [30,121,122]. The silica fume’s small particle size plays an important role in filling microscopic pores and improving bonding with fibers [123,124]. For the structural-light and ultra-light mixes, additional water was added to account for the water absorbed by the high number of pores in the aggregates. The mixes all utilized a high range water reducer (HRWR) (MasterGlenium 7920, Cleveland, OH, USA) at 756 mL/kg of cement. The HRWR helps to increase the workability of the concrete while maintaining the same water content. For the concrete mix designs incorporating fibers, the fiber volume fraction was set to 2% to strike a good balance between workability and tensile strength, based on a study conducted by Park, Lee, and Lim [123]. For the high-strength and ultra-light concrete mixes, the cementitious material content was set to 30% of the total volume of the mix. For the structural-light concrete mix, the cementitious volume was set to 20% in order to further reduce the density of the mix. 

**Figure 3:**
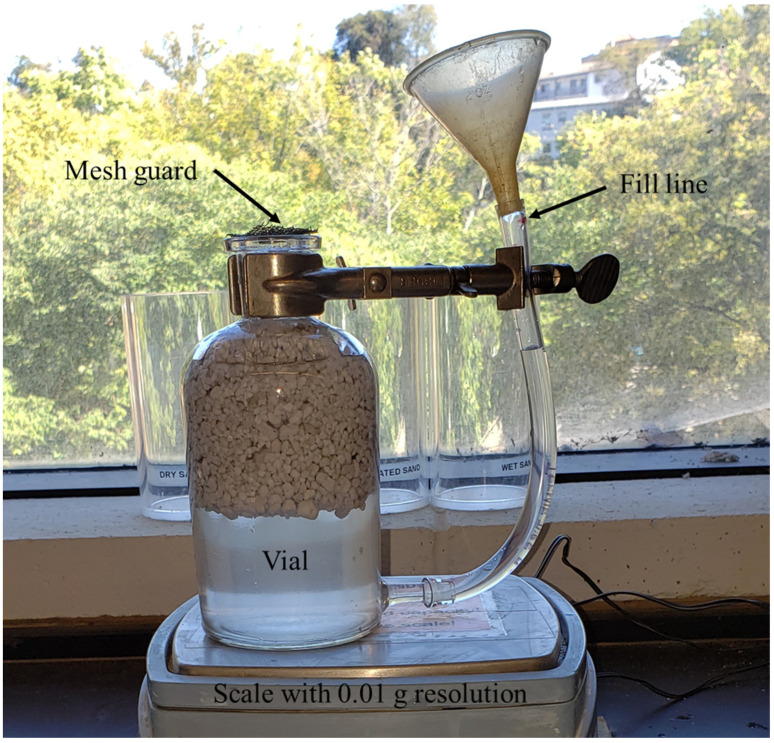
Experimental apparatus for density measurements of aggregates lighter than water by submerging aggregates in water.

Three different aggregate types were used for each of the three concrete mix designs and were the primary factor affecting the density of the final resulting concrete mixes. All of the aggregates were classified as fine aggregates to help reduce potential stress concentrations caused by large aggregates. M sand or crushed sand with a measured density value of 2.71 g/cm^3^ based on ASTM C128 was used for the high-strength concrete mix design [125]. Stalite (Stalite washed MS16 fines, Salisbury, NC, USA), an argillite slate aggregate with a density of 1.69 g/cm^3^, was used for the structural-light concrete design mix [126]. Perlite (Vigoro Organic Perlite soil amendment) was used for the ultra-light concrete mix design. The measured density of the perlite was 0.37 g/cm^3^ using a modified procedure of ASTM C128 [125]. As the perlite aggregates have a lower density than water, a modified procedure was performed in order to keep the perlite submerged in water. First, the aggregates were sieved using a number 20 (0.841 mm) sieve, and any material passing through the sieve was discarded to remove dust and fine particles. The aggregates were then placed in a glass vial covered with a no. 30 (0.595 mm) mesh screen. The mesh was weighted down with a small piece of metal over the top such that air could escape from the vial, but the aggregate could not. A clear plastic tube, approximately 10 mm in diameter, was attached to the bottom of the vial and carefully marked with a datum reference to ensure a repeatable volume of water was used for each measurement. The vial was then filled with water to the datum reference fill line and weighted with and without the aggregates in the vial, and the density was calculated based on the measurements in accordance with ASTM C128 [125]. Figure 3 shows the apparatus used to measure the density of perlite aggregates.

### 2.6. Concrete Mixing Procedure

It is important to note that the neat concrete mix design was used as a control for each concrete type in this study, thereby reducing variability when adding steel fiber and rCF for the other two mix designs. The same method was used to mix the high-strength and structural-light concrete mixtures. The silica fume and Portland cement were mixed together in a 7.6 L Hobert tabletop mixer for 1 min. The water and HRWR were added to the mixture slowly as the mixer was operating and were mixed for 2 min to promote optimal fluidity of the mix and reduce the cementitious materials from clumping. The aggregates were added to the mixture and mixed for an additional 3 min. Finally, for the fiber-reinforced mixes, the fibers were added and mixed for 5 min. For the neat concrete mix designs, the mixture was mixed for 5 min to ensure each batch received the same total mixing time.

The ultra-light mix was prepared in a similar manner as described for the high-strength and structural-light concrete mix designs. The dry cementitious material mixing, water, HRWR addition, and mixing steps were also the same as for the high-strength and structural-light concrete mixes. However, instead of adding the aggregate next, for the fiber-reinforced mixes the fibers were added and mixed for 5 min. As with the neat high-strength and structural-light mixes, the neat ultra-light mixture was mixed for 5 min to ensure consistent mixing times for all batches. For the last step, the aggregates were added to the mix and mixed for 3 min. The aggregates were added last to reduce potential damage to the relatively fragile perlite aggregates from over-mixing. For all the concrete batches, a flow test in accordance with ASTM C1437-20 was performed immediately after mixing to evaluate and compare their rheology [127].

### 2.7. Casting Samples

**Figure 4:**
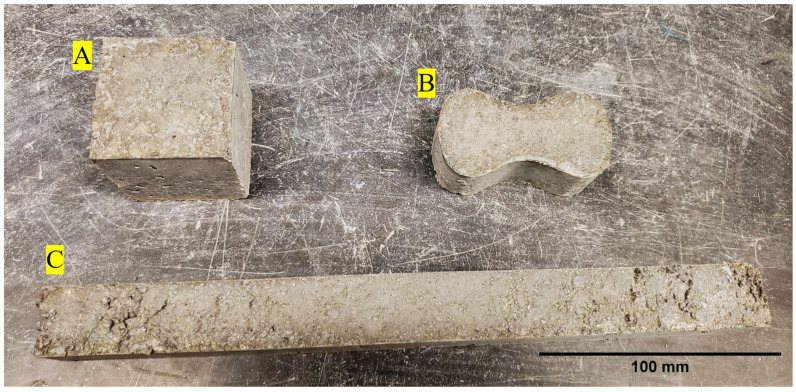
Example (**A**): compression cube, (**B**): tension briquettes, and (**C**): flexural beam for mechanical testing.

Three types of samples, namely tension briquettes, compression cubes, and flexural beams, for all nine concrete mix designs, were cast for mechanical characterization as shown in Figure 4. The steel or brass molds for each sample type were first filled halfway with concrete and then tamped down using a 10 mm by 25 mm wooden tamper. Care was taken to ensure that the entire surface of the concrete was tamped. The molds were then filled to capacity and tamped again. The sides of the molds were tapped with a rubber mallet to help release air pockets. The surfaces of the samples were then smoothed and flattened with a straight edge. For the neat and steel-fiber-reinforced samples, care was taken to hand mix the concrete as it was being molded to ensure each sample was representative of the whole mix since the high flow of the mixes could cause the aggregates and cement to separate.

### 2.8. Mechanical Testing of Casted Samples

**Figure 5:**
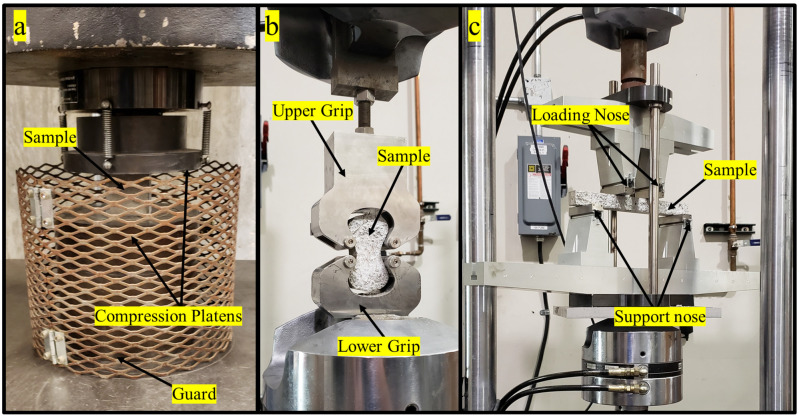
Mechanical testing experimental setup for (**a**): compression cube, (**b**): tensile test, and (**c**): flexural test with a 229 mm major span and a 76 mm minor span.

The concrete samples were evaluated for compression, tensile, and flexural strength as summarized in Table 3 and Figure 5. Figure 5a shows the experimental setup for compression testing where the compression strength was evaluated in accordance with ASTM C109, where the samples were monotonically loaded on a 600 kN load test frame (Instron, Norwood, MA) to mechanical failure. The samples should be loaded at a rate of 900–1800 N/s according to the ASTM C109 standard. However, as the testing load frame had insufficient force control, it was determined to use a displacement control at a rate 0.1 mm/min to provide consistent results meeting the ASTM C109 specifications with the required force rate [128]. The tensile tests were performed in accordance with ASTM C307, and the test setup is shown Figure 5b. Using a 100-kN MTS servo hydraulic load frame, the tension briquette samples were mounted into custom-made stainless steel grips using ASTM specifications and loaded monotonically at a crosshead rate of 2 mm/min until mechanical failure [111,129]. The flexural samples were tested in accordance with ASTM C947, and Figure 5c shows the experimental setup with a four-point bending fixture (Wyoming Test Fixtures model WTF-LF, Salt Lake City, UT, USA) [130]. The samples were loaded monotonically at a crosshead rate of 1.27 mm/min to mechanical failure on a 100-kN MTS load frame [131]. The test deviated from the ASTM C947 standard where the 25 mm depth × 25 mm width × 305 mm length samples were cast as previously described instead of being cut from a sheet of concrete [131]. The force and displacement values for all the test samples were collected at a rate of 5 Hz [111].

### 2.9. Micro X-ray Computed Tomography

In order to examine the microstructure of the concrete mix designs, a custom-developed µ-XCT machine consisting of a four-axis (x, y, z, θ) rotary stage (Aerotech, Pittsburgh, PA, USA) was used to mount and scan nine samples cored from the tensile briquette sample (approximately 18 mm in diameter by 25 mm in length). Each sample was scanned over an angular range of 360 degrees with a voxel resolution of approximately 12 µm using a voltage of 150 kV and amperage of 137 µA (Hamamatsu L8121-03, Shizuoka, Japan). A total of 3001 12-bit 2D projections (2316 × 2316 pixels) were collected and normalized, and sinograms were obtained to reconstruct 2D slices using a commercial reconstruction algorithm (Octopus 8.9.3, Ghent University, Ghent, Belgium). Additionally, artificial artifacts such as ring artifacts and beam hardening were reduced using correction methods applying the aforementioned reconstruction algorithm [111,132]. Each 2D-reconstructed slice consisted of a grayscale value between a maximum pixel value of 65,535 (white pixel), corresponding to the densest region of the material, and a minimal pixel value of 0 (black pixel), corresponding to the least dense region, such as air [133,134]. The 3D volume visualization was obtained from the 2D-reconstructed slices, where these slice images were post-processed for quantitative data using a commercial 3D visualization software (ScanIP, Simpleware U-2022.12 Build 325). To characterize the concrete mix designs for porosity, each 3D volume was cropped to approximately 16.5 mm diameter by 14.88 mm height. The image data sets for each sample were resampled from 12 µm to 30 µm to reduce the computational image processing time. Additionally, it must be noted that individual rCF fibers were difficult to detect at 30 µm resolution; however, larger bundles of the fibers could be identified within the concrete mix designs obtained using µ-XCT. 

An upper- and lower-threshold grayscale value was applied manually as a first step in image segmenting to create masks for the concrete, pore (air), and fiber phases within the reconstructed 3D volume. To further enhance the segmenting, concrete and voids geometries flood-fill algorithms were applied. Overlapping masks were subtracted to unambiguously identify concrete and void phases within the 3D reconstructed volume using Boolean operations [135]. The 2D reconstructed slices for each volume were carefully inspected for overlapping phases or masks. It must be noted that the fiber phases were segmented only for steel-fiber-reinforced concrete mix design samples because the grayscale value contrast between the steel fiber and concrete phase was high. Although the rCF-reinforced fiber phase had similar neighboring grayscale values to the concrete phases, bundles of the rCF were identified within the 3D volumes and are discussed later in this study [111]. Hence, an investigation of the microstructure of the concrete and voids phases in the concrete mix designs was the primary aim for this study.

### 2.10. Thermal Conductivity

**Figure 6:**
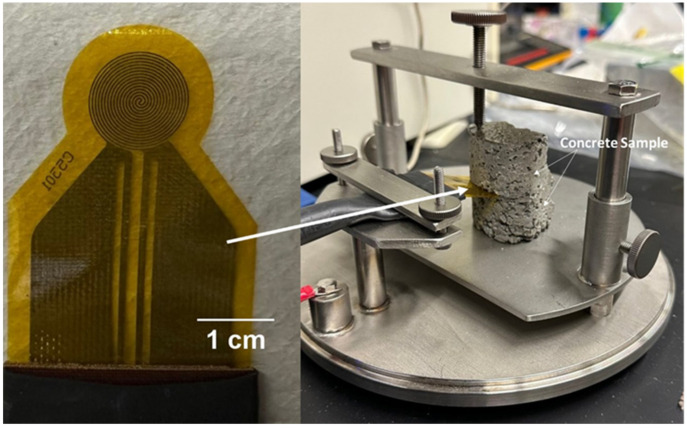
Thermal conductivity measurement experimental setup using the transient plane source (TPS) approach with the (**left** image) Hot Disk^®^ sensor (**right** image) positioned between two concrete parts of the tensile sample.

In this study, the thermal conductivity of the mixes was measured to investigate how porosity and different fibers affected the concrete mix design. Thermal conductivity measurements were performed on two halves of tensile briquettes for each concrete mix design using a transient plane source instrument (Hot Disk^®^ TPS 2500 S, Göteborg, Sweden) consisting of a 6.403 mm radius Kapton sensor in accordance with ISO 22007-2 [136]. To obtain accurate results, prior to performing thermal conductivity measurements, each surface in contact with the sensor was ground using silicon carbide grinding paper (CarbiMet PSA, 180 [P180], Buehler, Lake Bluff, IL, USA), followed by 320-grit silicon carbide grinding paper (CarbiMet PSA, 320 [P400], Buehler, Lake Bluff, IL, USA) on a grinder-polisher (Buehler MetaServ 250). The samples were then dried in the oven at 80 °C for approximately 2 h to remove moisture. Figure 6 shows an example of the sensor setup positioned between the samples where measurements were conducted at room temperature. It must be noted that a sample holder hood was used to cover the sample before each measurement to minimize any environmental effects and any air flow around the samples [137]. According to Hot Disk^®^, an electrical current is passed through the sensor to the samples with sufficient current to generate heat up to several degrees, and it records the resistance or temperature as a function of time. This temperature change rate of thermal transport from the sensor to the materials is “highly dependent on thermal transport properties of the surrounding material” [110,137,138]. 

According to Hot Disk^®^, the thermal conductivity measurement is based on the following theoretical relationship shown in Equation (3) for a time-dependent temperature increase for the Transient Plane Source technique:

(3) $$∆T_{ave}(\tau )=\frac{P_{0}}{\pi^{3/2}\times \alpha \times ᴧ}\times D(\tau )$$

the total output power *P*_0_ from the sensor, *α* represents the overall radius of the disk, *D*(*τ*) is a dimensionless time dependent function, and ᴧ represents the thermal conductivity of the tested samples. The *D*(*τ*) function has the following relationship

(4) $$\tau =\sqrt{\frac{t}{\Theta }}$$

where *t* represents the measured time from the start of the transient recording. The characteristic *Θ* is defined as the following:

(5) $$\Theta =\frac{\alpha^{2}}{\kappa }$$

where *κ* represents the sample’s thermal diffusivity. A computation plot is generated of the recorded temperature increase versus *D*(*τ*) to obtain a straight line, where $\frac{P_{0}}{\pi^{3/2}\times \alpha \times ᴧ}$ is the slope and Δ*T_i_* is the intercept. Due to both *Θ* and *κ* are not known prior to the experiment, the thermal conductivity is calculated through a iterative process [137].

### 2.11. Statistical Analysis

Statistical analysis was performed for each concrete mix design to compare the measured physical, mechanical, and thermal conductivity properties. The average and standard deviation values were calculated for the flow, compression, tension, and flexural properties, in accordance with ASTM standards. The average and standard deviation values were calculated for the thermal conductivity properties. Since only two density measurements were obtained for each concrete mix design, the two density values were reported. It must be noted that density values are averaged for the Results and Discussion portions of this study to compare the density for each concrete mix design. 

## 3. Results

### 3.1. Scanning Electron Microscopy and Element Chemical Composition of Fibers

**Figure 7:**
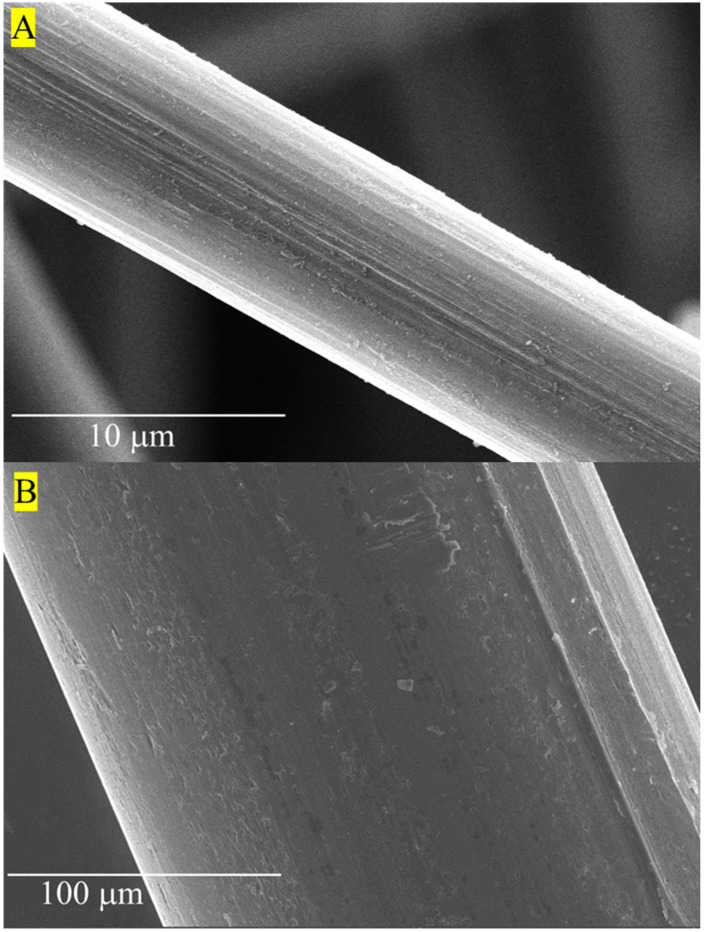
Scanning electron microscopy of (**A**) recycled carbon fiber, and (**B**) steel fiber.

**Figure 8:**
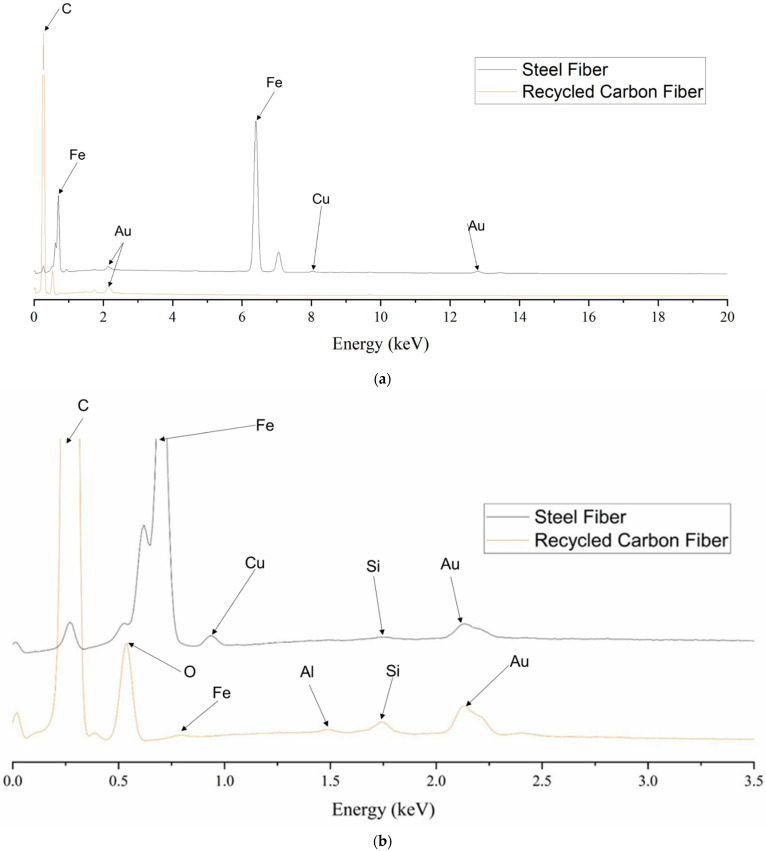
Energy dispersive X-ray spectra of (**a**) steel fiber and recycled carbon fiber, identifying the chemical elemental constituents for each fiber, (**b**) with inset showing element constituents for a binding energy range of 0 to 3.5 keV.

Figure 7 shows example surface morphologies for both rCF and steel fibers. The steel fiber exhibited longitudinal grooves from the manufacturer’s drawing process. Similarly, the surface roughness and longitudinal grooves of the rCF are indicative of the precursor fiber type, fiber spinning, and converting from precursor fiber to carbon fiber. Figure 8 shows the EDS spectra for rCF and steel fiber. The elemental chemical composition of the steel fibers consisted of carbon (C), iron (Fe), and copper (Cu). Strong C and O peaks were observed for the rCF, where the O peak corresponds to the oxidation from complex chemical reactions of converting a carbon fiber precursor to carbon fiber [139]. Additionally, the presence of Si suggests that residual polymer fiber sizing is present on the surface of the rCF [111,140]. Moreover, the peaks observed for Fe and aluminum (Al) detected for the rCF suggest that the fibers were exposed to impurities during handling and pyrolysis in the recycling process. 

### 3.2. Wide Angle X-ray Scattering of Carbon fibers

Table 4 summarizes the WAXS for rCF compared to the reference commercial carbon fiber T700 carbon fiber. Both batches of rCF had similar d-spacings and crystalline parameters. Furthermore, the rCF fibers had similar results to those of T700 carbon fiber with the exception that the rCF had smaller crystalline dimensions. The WAXS results suggest that rCF has a comparable crystalline structure to the industrial carbon fiber T700 and possesses suitable mechanical properties as a fiber reinforcement in concrete mix designs.

### 3.3. Mechanical Properties of the Fiber-Reinforced Concrete Mix Designs

Table 5 summarizes the comparison of mechanical properties, flow properties, and density of the concrete mixes. The steel fibers and neat concrete mix design were used as a baseline measurement as a comparison to the rCF-reinforced concrete mix designs. For the high-strength concrete, the compressive strength of the rCF mix design (77.0 MPa) was approximately 16.3% lower than the neat mix design (90.7 MPa) and approximately 13.4% lower than the steel fiber mix design (88.1 MPa). The tensile strength of the rCF mix design (4.55 MPa) was approximately 0.7% higher than the neat mix design (4.52 MPa) and about 34.2% lower than the steel fiber mix design (6.43 MPa). The flexural strength of the rCF mix design (9.40 MPa) was approximately 21.0% higher than the neat mix design (7.61 MPa) and approximately 88.4% lower than the steel mix design (24.28 MPa). The flow of both the steel and neat mix was 150+%, with rCF having a flow of 25%. The average density of the rCF mix design (2.23 g/cm^3^) was approximately 4.8% lower than the neat mix design (2.37 g/cm^3^) and approximately 6.1% lower than the steel mix design (2.37 g/cm^3^). For the structural-light concrete, the compressive strength of the rCF mix design (28.4 MPa) was approximately 73.2% lower than the neat mix design (61.2 MPa) and approximately 20.8% lower than the steel mix design (35.0 MPa). The tensile strength of the rCF mix design (2.90 MPa) was approximately 14.8% higher than the neat mix design (2.50 MPa) and approximately 48.8% lower than the steel mix design (4.77 MPa). The flexural strength of the rCF mix design (2.57 MPa) was approximately 82.8% lower than the neat mix design (6.20 MPa) and approximately 118.3% lower than the steel mix design (10.02 MPa). The neat mix had the highest flow, at 84.4%, followed by the steel fiber mix, with a flow of 20.3%. Unfortunately, due to low workability and poor cohesion in the concrete mix, the rCF mix design crumbled during the test, leading to an invalid test or an effectively 0% flow. The average density of the rCF mix design (1.53 g/cm^3^) was approximately 10.5% lower than the neat mix design (1.70 g/cm^3^) and approximately 15.7% lower than the steel mix design (1.79 g/cm^3^). For the ultra-light concrete, the compressive strength of the rCF mix design (21.7 MPa) was approximately 13.7% lower than the neat mix design (24.9 MPa) and approximately 62.4% lower than the steel fiber mix design (41.4 MPa). The tensile strength of the rCF mix design (3.21 MPa) was approximately 72.6% higher than the neat mix design (1.50 MPa) and approximately 58.6% lower than the steel fiber mix design (5.87 MPa). The flexural strength of the rCF mix design (5.62 MPa) was approximately 48.7% higher than the neat mix design (3.42 MPa) and approximately 97.5% lower than the steel fiber mix design (16.32 MPa). The flow of both the neat and the steel fiber was 150+%, with rCF having a flow of 1.6%. The average density of the rCF mix design (1.48 g/cm^3^) was approximately 2.0% lower than the neat mix design (1.51 g/cm^3^) and approximately 12.7% lighter than the steel fiber mix design (1.68 g/cm^3^). 

**Figure 9:**
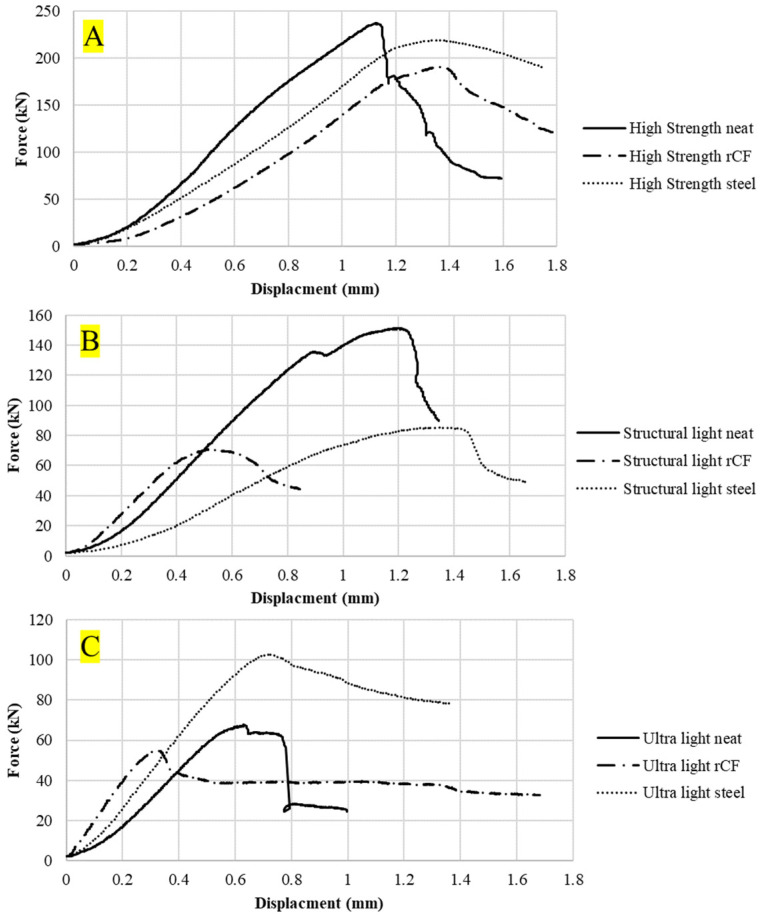
Compression force–displacement curves of the compression concrete samples for the (**A**): high-strength, (**B**) structural-light, and (**C**) ultra-light mixes.

**Figure 10:**
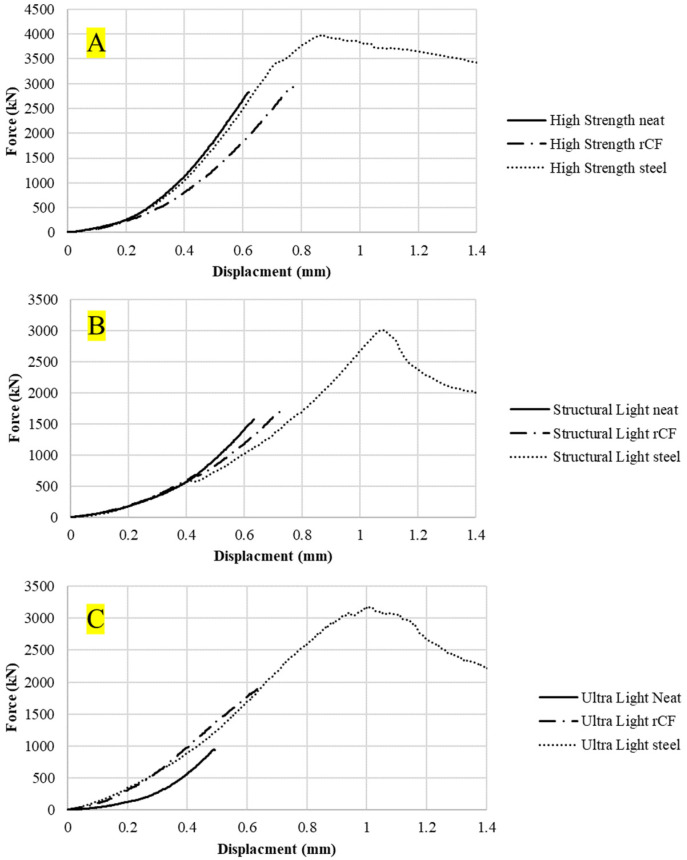
Tensile force–displacement curves of the tensile briquette concrete samples for (**A**): high-strength, (**B**) structural-light, and (**C**) ultra-light mixes.

**Figure 11:**
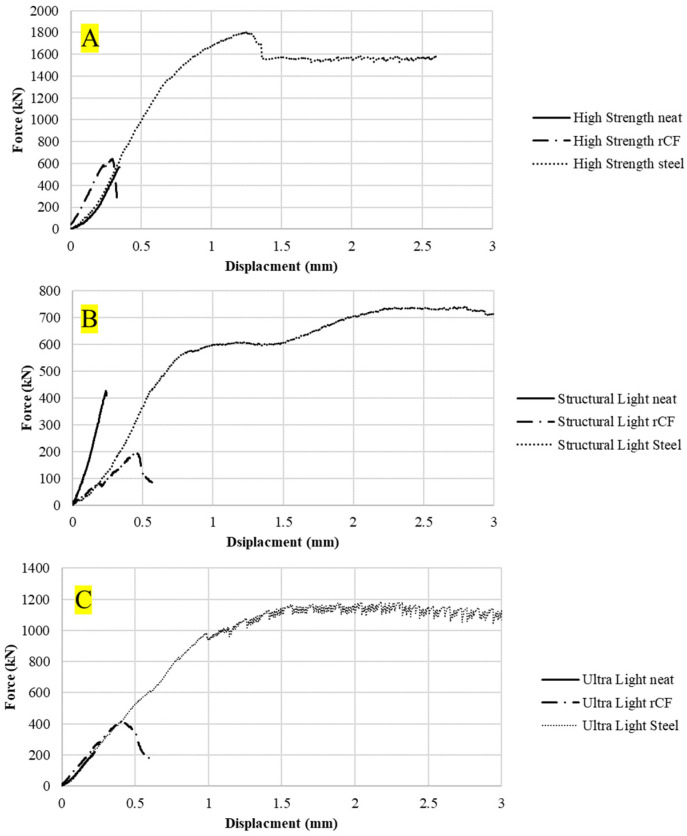
Flexural force–displacement curves of the flexural concrete sample for (**A**) high-strength, (**B**) structural-light, and (**C**) ultra-light mixes.

Figure 9 shows the compression force–displacement curves for an example sample for each concrete mix design. The neat mix had a minimal force-carrying capacity or ductility after reaching its maximum force whereas the rCF and steel-reinforced samples showed a small amount of ductility. Figure 10 shows the tensile force–displacement curves for the different concrete mixes. It can be observed that both the neat and rCF samples have no ductility after peak force whereas the steel samples exhibited a considerable amount of ductility. Figure 11 shows the flexural force–displacement curves for the concrete mixes. Similar to the tensile results, the rCF and neat samples had minimal post-peak-force ductility whereas the steel fiber exhibited a significant amount of ductility. It is also interesting to note the failure behavior of the flexural high-strength and ultra-light steel-fiber-reinforced samples where, beyond the peak force, the force seems to oscillate up and down. This behavior can be attributed to the fiber pull-out mechanisms of the steel fibers. The steel fibers gradually pull out of the concrete due to the friction bond between the concrete and fiber, resulting in a repeated catch and slip failure mechanism which, in turn, leads to a jagged force verses deflection curve. In contrast to the steel-fiber-reinforced flexural samples, the rCF exhibited rupture failure behavior, showing that rCF-reinforced concrete mix designs in this study are not ductile.

### 3.4. Micro X-ray Computed Tomography

**Figure 12:**
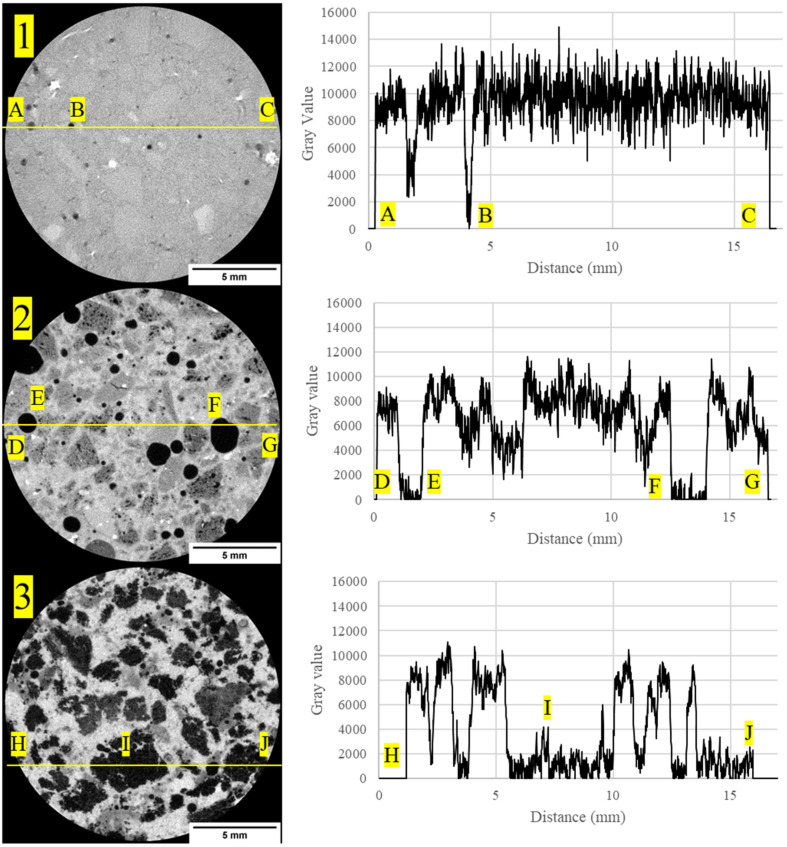
(Left column) The 2D-reconstructed cross-section of different concrete samples designated as 1: high-strength concrete, 2: structural-light concrete, 3: ultra-light concrete. (Right column) Corresponding line profiles showing grayscale intensity values based on a minimum value of 0 (black pixel), and where 65,535 (white pixel) corresponds to the densest region. Key microstructural features of interest, porous air void regions, are designated by points (**A**–**J**) and shown spatially across the 2D cross-section in the left column.

**Figure 13:**
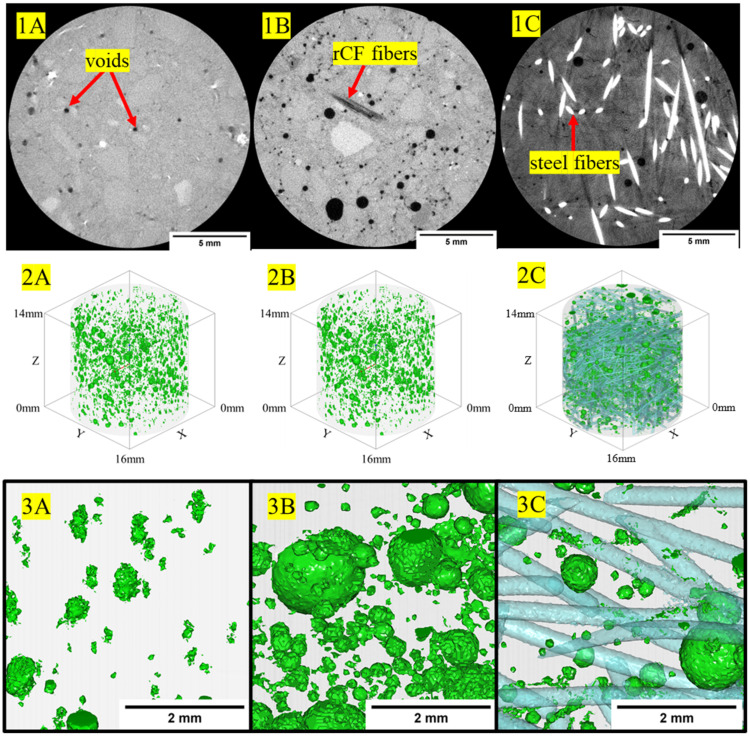
(**1A**–**1C**) The 2D-reconstructed cross-sections showing fiber bundle phases, air voids, within the high-strength host concrete. (**2A**–**2C**,**3A**–**3C**) The 3D-reconstructed volume visualization of voids spatially within the high-strength host concrete. Note: (**1C**,**2C**,**3C**) fiber phase for steel fiber is shown.

**Figure 14:**
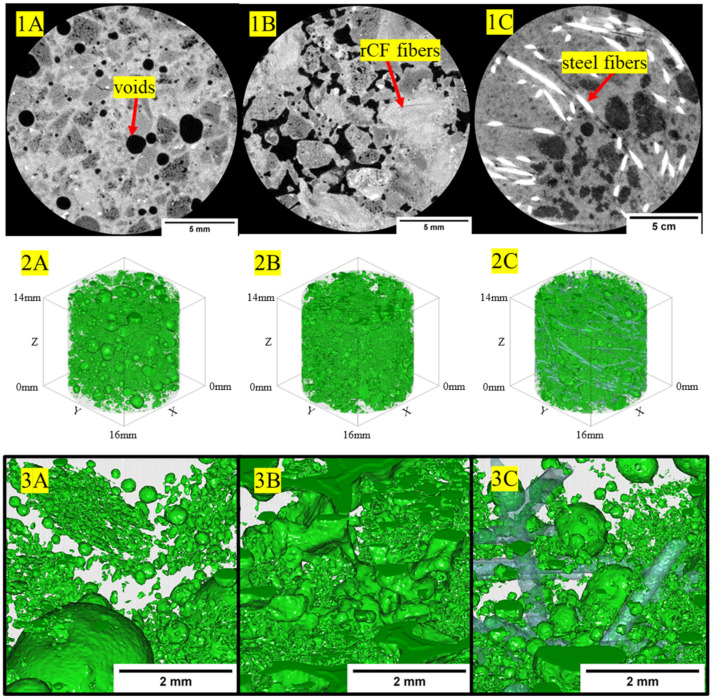
(**1A**–**1C**) The 2D-reconstructed cross-sections showing fiber bundle phases, air voids, within the structural-light host concrete. (**2A**–**2C**,**3A**–**3C**) The 3D-reconstructed volume visualization of voids spatially within the host concrete. Note: (**1C**,**2C**,**3C**) fiber phase for steel fiber is shown.

**Figure 15:**
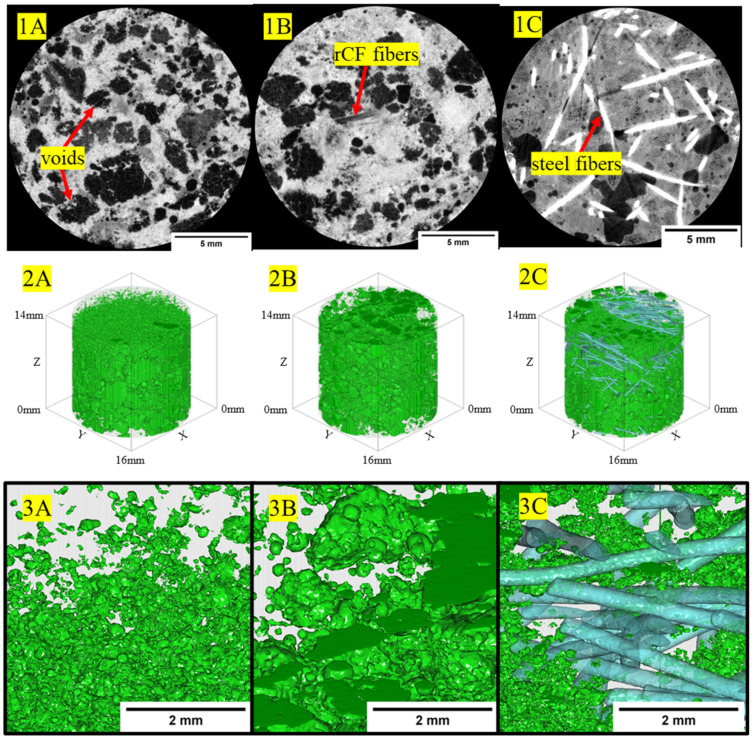
(**1A**–**1C**) The 2D-reconstructed cross-sections showing fiber bundle phases, air voids, within the ultra-light host concrete. (**2A**–**2C**,**3A**–**3C**) The 3D-reconstructed volume visualization of voids spatially within the host concrete. Note: (**1C**,**2C**,**3C**) fiber phase for steel fiber is shown.

Figure 12 shows example 2D-reconstructed slices for neat high-strength, structural-light, and ultra-light concrete mix designs. The high-strength concrete exhibited the densest regions within the concrete phase of the sample with grayscale values having a baseline value of approximately 9000, whereas the less dense structural-light and ultra-light both had baseline grayscale values of 8000 for the concrete matrix phase. Additionally, the pores, or least dense regions, where the grayscale values approach zero, are more frequent and visible in the structural-light and ultra-light than in the high-strength concrete. These grayscale differences for the concrete phase can be attributed to greater X-ray attenuation of the denser neat high-strength concrete mix (average: 2.34 g/cm^3^) than in the neat structural-light (average: 1.70 g/cm^3^) and neat ultra-light (average: 1.51 g/cm^3^) mixes. Additionally, unambiguous detection of the microstructure granularity within the concrete phases and pore phases within the 2D-reconstructed cross-section for each concrete mix design demonstrates the effectiveness of this µ-XCT technique. Figure 13, Figure 14 and Figure 15 show example 2D-reconstructed cross-sections and 3D-reconstructed volumes highlighting the segmented pores within the cored samples used in this study for the neat, rCF-reinforced, and steel-fiber-reinforced concrete mix designs (high-strength, structural-light, and ultra-light). The details of the microstructure of the pore formation and distribution spatially can be clearly observed for each concrete mix. Table 6 summarizes the void volume content for each concrete mix, where, considering all mix designs, the high-strength concrete had the lowest porosity (0.9–4.7%), followed by an increase in voids for structural-light (12.2–16.2%), with the highest void content observed for ultra-light (27.7–37.1%). This void volume, calculated using the 3D visualization software, agrees visually with the spatially sparser voids observed for the high-strength concrete (Figure 13(2A–2C,3A–3C)), compared to the presence of significantly more voids observed spatially for the structural-light (Figure 14(2A–2C,3A–3C)) and ultra-light concretes (Figure 15(2A–2C,3A–3C)). 

Additionally, it is interesting to note the interconnectivity of the pores observed for the structural-light and ultra-light concrete mix designs. Due to the interconnectivity of voids and the segmentation procedure used in this work, individual voids could not be quantified for a pore size distribution. However, this demonstrates the effectiveness of using the µ-XCT technique to obtain void volume content spatially and quantitative information including the effects of the fiber reinforcement of the concrete. As shown in Table 6, incorporating fiber reinforcement increased the void volume content for the high-strength concrete and structural-light concrete mix designs. Notably, with respect to the neat high-strength concrete void volume content (0.9%), the incorporation of rCF (4.7%) into the concrete mix had the highest effect, with a 3.8% increase in void volume content compared to the steel fiber (1.4%). Similarly, with respect to the neat structural-light void volume content (12.2%), the incorporation of rCF (16.2%) had the highest effect with an increase of void volume content by 4% compared to the steel fiber (14.9%). Conversely, the void volume decreased with fiber reinforcement for the ultra-light concrete mix design, where, with respect to the neat ultra-light (37.1%), the steel fiber (30.7%) decreased the void volume content by 7% compared to a higher decrease (9.4%) observed in the rCF (27.7%).

### 3.5. Thermal Conductivity of Fiber-Reinforcement Concrete Mix Designs

Table 7 summarizes the thermal conductivity for the high-strength, structural-light, ultra-light neat, steel-fiber-reinforced, and rCF-reinforced mixes. Considering all mix designs, the high-strength concrete had the highest thermal conductivity (1.502–1.666 W/mK), followed by the structural-light (0.551–0.752 W/mK), and the lowest thermal conductivity was observed for the ultra-light (0.341–0.535 W/mK). With respect to the neat high-strength concrete (1.666 W/mK), the incorporation of rCF (1.502 W/mK) had a lower thermal conductivity (10.4%), and the steel fiber mix (1.787 W/mK) had a higher thermal conductivity (7%). Similarly, with respect to the neat structural-light concrete (0.752 W/mK) the incorporation of rCF (0.551 W/mK) resulted in lower thermal conductivity (30.9%), and the steel fiber mix (0.945 W/mK) had a higher thermal conductivity (22.7%). Notably, with respect to the neat ultra-light concrete (0.341 W/mK), the incorporation of rCF (0.535 W/mK) had the highest thermal conductivity (44.3%), and the incorporation of the steel fiber (0.515 W/mK) had a lower increase in thermal conductivity (40.7%) than the neat concrete.

### 3.6. Scanning Electron Microscopy of Failed Concrete Mix Designs

**Figure 16:**
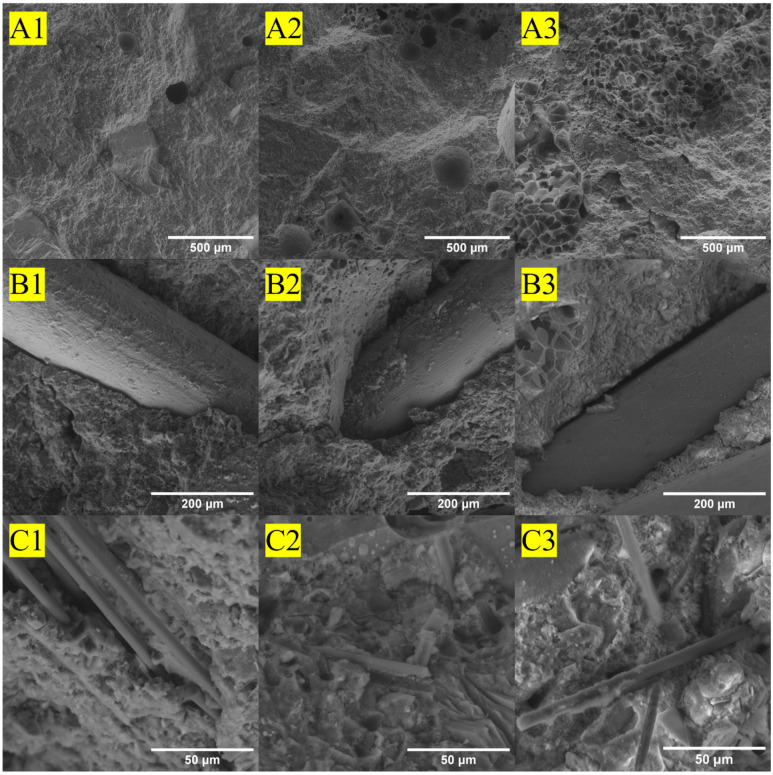
SEM images of the failed tension samples. (**A1**): neat high-strength mix, (**A2**): neat structural-light mix, (**A3**): neat ultra-light mix, (**B1**): steel high-strength mix, (**B2**): steel structural-light mix, (**B3**): steel ultra-light mix, (**C1**): rCF high-strength mix, (**C2**) rCF structural-light mix, (**C3**) rCF ultra-light mix.

The mechanically failed tensile samples for each of the nine different concrete mix designs were examined using SEM to investigate the failure mechanisms for the fibers and surrounding host concrete matrix, as shown in Figure 16. Figure 16A1 shows the fracture surface of the neat tensile sample with minimal voids and a dense concrete matrix. Figure 16A2 shows the structural-light mix; note the sponge-like structure of the expanded shale aggregate in the top right corner and the higher number of pores than in the high-strength mix. Figure 16A3 shows the ultra-light mix, demonstrating the highly porous structure of the perlite aggregate in the bottom left and top right corner and the larger number of pores than those found in either the high-strength or structural-light mixes. Additionally, for the neat concrete mix designs (Figure 16A1–A3), an increase in the presence of voids can be clearly observed from the high-strength, with minimal voids, followed by structural-light, with an increased presence of voids, to a significant presence of voids in the ultra-light, agreeing well with void volume content trends observed in Table 6, µ-XCT 2D-reconstructed slices, and 3D-reconstructed volumes (Figure 12, Figure 13, Figure 14 and Figure 15). For each of the steel fiber-reinforced mixes (Figure 16B1–B3), it can be seen that the steel fibers are relatively clean and show a minimal bonding concrete matrix. Figure 16C1–C3 show the rCF-reinforced mixes, and it can be observed that there is fiber clumping and minimal concrete adhering to the fibers, indicating poor bonding between the concrete and fibers similar to that seen with steel fibers.

## 4. Discussion

Investigation of the structure–process–property relationships between the rheological behavior, porosity, and influence of rCF on the mechanical performance for all the considered concrete mix designs was the primary aim of this study. The SEM surface morphology (Figure 7) and EDS elemental composition (Figure 8) confirmed minimal polymer sizing was present on the surface of the rCF. Furthermore, crystalline parameters for rCF (Table 4) were comparable to those of a high-performance commercial carbon fiber. Thus, the combination of SEM, EDS, and WAXS suggests that rCF have suitable physical and mechanical integrity for incorporation into concrete mix designs for structural applications. 

It can be observed in Table 5 that, with regards to both tension and flexural strength values, the rCF mix designs were lower than steel fiber but higher than neat concrete with the one exception being the structural-light flexural strength. It is hypothesized that the poor results for the structural-light mix were due to the negligible flow of the mix. The lack of flowability led to samples with more voids and less consistency as the concrete was unable to adequately consolidate into the molds. The structural-light mix’s low flow was due to the reduction in cementitious material content by 20% volume compared to the 30% by volume of the other mix designs, as can be seen in Table 2. The effects of the reduced cementitious material content on the flow of the concrete were seen for both the neat (84.4%) and steel (20.3%) samples; however, the addition of rCF worsened the issue. It was observed across the high-strength, structural-light, and ultra-light mixes that the addition of rCF negatively affected the flow of the concrete. The probable reason for rCF reducing flow is dispersion, where the carbon fibers have a smaller fiber diameter (6.7 µm), a higher aspect ratio (224), consisting of significantly more individual carbon fibers than steel fiber with a 200 µm fiber diameter, and a smaller aspect ratio (65). For the rCF, this increases the fiber surface area that needs to be wetted by the water and allows for fibers to entangle more frequently during mixing. Furthermore, the fibers can only be mixed into the cement matrix portion of the concrete; thus, for the structural-light mix there was less volume for the fibers to disperse into, creating compounding issues that led to a mix with negligible flow. It is therefore important to consider not just the volume fraction of fibers based on the total concrete volume, but also the fiber volume fraction of fibers based on the cementitious material volume. However, it can be observed, based on the high-strength and ultra-light mixes, that rCF reinforcement is an effective method of increasing the tensile (Figure 10A,C) and flexural strength (Figure 11A,C) of neat concrete when the flow of the concrete is maintained. This was also observed in Patchen, Young, and Penumadu, where the addition of rCF to ultra-high performance concrete led to an increase in tensile strength [111].

It can be seen in Table 5 that the rCF-reinforced concrete mix design was the least dense of each of the three primary mixes, followed by the neat and then the steel fiber. It was expected that the steel-fiber-reinforced mixes would be densest because the steel fibers (7.8 g/cm^3^) are much denser then the aggregates (2.71–0.37 g/cm^3^) or the rCF (1.81 g/cm^3^) they are replacing. The ultra-light mix illustrates this the best as the steel-fiber-reinforced mix (1.68 g/cm^3^) was 0.2g/cm^3^ denser than the rCF-reinforced mix (1.48 g/cm^3^). Therefore, rCF reinforcement could be ideal in applications where the density of the concrete is critical. It should be noted that while the rCF-reinforced structural-light mix (1.53 g/cm^3^) had a much lower density than the other structural-light mixes (1.70–1.79 g/cm^3^), this was mostly because the poor flow traps air and creates voids during mixing and casting. 

Table 5 shows that compressive strength of neat concrete had the highest strength values for both the high-strength mixes (90.7 MPa) and the structural-light mix concrete designs (61.2 MPa). However, for the ultra-light mix, the steel-fiber-reinforced mix had the largest compression strength (41.4 MPa). This can be attributed to the fact that in the ultra-light mix, the perlite aggregate’s low-density characteristic makes it light enough to float to the surface of the concrete in the neat mix. Conversely, the steel fibers help to constrain the aggregates by reducing flow and trapping them in the fibers, allowing for more structurally consistent samples to be mixed and cast while maintaining excellent flow and consolidation. For the structural-light mix, the reduction in compressive strength is significant at approximately half the strength of the neat mix (61.2 MPa) for both the steel fiber (35.0 MPa) and rCF (28.4 MPa) mixes. This is, again, because the low flow causes an increase in voids volume within the concrete mix and poor consolidation of the concrete, further illustrating the importance of adequate flow and adjusting the fiber loading based on the cement content to maintain flow. 

Figure 9, Figure 10 and Figure 11 show example force–displacement curves for the different mixes and reinforcement types. Overall, it was observed that steel fiber provides far superior ductility to neat concrete and rCF concrete. Additionally, it was observed with the flexural test how the steel fibers provided higher ductility. Instead of rupturing like rCF, the steel fibers gradually pulled out of the concrete when loaded with tensile forces. This is best illustrated in Figure 11C. After the peak strength is observed, the flexural-force–displacement curve has a saw-tooth pattern: Where the steel fibers undergo frictional debonding during mechanical failure (i.e., they pull out of the concrete then catch, causing a small amount of drop in the force load and then an increase in the loading to a maximum or spike in the load in a repeating pattern), this saw-tooth curve results. The ductility of concrete can be critical in structural applications and is one of the areas that requires optimizing when future studies consider incorporating rCF.

Table 6 shows porosity data calculated from the µ-XCT technique showing that the void content of the mixes increased from the high-strength (0.9–4.7%), followed by structural-light (12.2–16.2%), with the highest porosity observed for the ultra-light mix (27.7–37.1%). This trend is due to the aggregates used in the structural-light and ultra-light mixes having a large number of air voids, increasing the overall void content of the concrete and reducing its density. Additionally, it can be observed that in the high-strength neat mix (0.9%) and structural-light neat mix (12.2%), the void content goes up with the addition of fibers with rCF (4.7% for high-strength and 16.2% for structural-light), causing a larger increase than the steel fiber (1.4% for high-strength and 14.9% for structural-light). For the ultra-light mix, the opposite trend was observed: The neat mix (37.7%) had the highest void content, followed by steel fiber (30.1%), and rCF (27.7%) had the lowest void volume. This result could be due to the perlite aggregate used in the ultra-light mix being light enough to float in the neat mix whereas, when fibers were included, these helped to constrain the aggregate and keep the mix more uniform through the depth of the sample. Additionally, it was detected that a majority of the void space was interconnected in the structural-light and ultra-light mixes. This is due to both a high porosity, and therefore inherently higher interconnection of the voids, and overlapping grayscale values around the edges of the voids because the concrete matrix and void are within the same pixel value range, which could cause the separate voids to blend together during the image segmentation analysis.

As shown by the thermal conductivity measurements presented in in Table 7, the high-strength mix (1.502–1.666 W/mK) had the highest thermal conductivity, followed by the structural-light (0.551–0.752 W/mK), and the ultra-light (0.341–0.535 W/mK) had the lowest thermal conductivity. This trend corresponds well to the void volume values obtained from the µ-XCT technique (Table 6), which show the inverse trend with increasing void content. This trend is expected because air is a significantly less effective thermal conductor than the host concrete matrix. Furthermore, both the steel fiber and rCF by themselves have a higher thermal conductivity than neat concrete, suggesting that the addition of fibers will increase the thermal conductivity of the composite mix. This can be clearly observed with the steel-fiber-reinforced high-strength (1.787 W/mK) and structural-light (0.945 W/mK) mixes having higher thermal conductivity than the corresponding neat (1.666 W/mK for high-strength and 0.752 W/mK for structural-light) mixes. For the rCF-based high-strength (1.502 W/mK) and structural-light (0.551 W/mK) mixes, a decrease in thermal conductivity was observed compared to the neat (1.666 W/mK for high-strength and 0.752 W/mK for structural-light) concrete. This is likely due to the increased void content of the rCF-reinforced mixes causing a decrease in thermal conductivity that outweighs the increase in thermal conductivity caused by the fibers. For the ultra-light mixes, the neat (0.341 W/mK) had the lowest thermal conductivity but also the highest void content (37.1%). The steel fiber and rCF had similar void content (30.1% for steel fiber and 27.7% for rCF) and thermal conductivity (0.515 W/mK for steel fiber and 0.535 W/mK for rCF), suggesting that for the same amount of voids, the different fiber types affect the thermal conductivity in a similar manner. Overall, it can be concluded that the void content is the primary factor affecting the thermal conductivity followed by the addition of fibers. However, the increase in thermal conductivity caused by the fibers can be outweighed by the additional voids created because the fibers reduce the workability of the concrete. This demonstrates the effectiveness of correlating the effect of void volume content within the rCF-reinforced concrete mix designs in this study using µ-XCT and thermal conductivity measurements.

Figure 16 shows the SEM images of the different mixes. For both the structural-light and ultra-light mixes, small voids within the aggregates can be seen that help to reduce the density of the concrete. It can be observed in the steel fiber mix that the fibers do not break, but instead pull out of the concrete, showing that they do not develop their full rupture strength. Likewise, it can be seen in the rCF mixes that while the fibers have been broken, they still pull out of the concrete, showing that the bonding between the fibers and concrete matrix is still not ideal. This can be confirmed by observing that very little concrete is bonded onto the rCF fibers. Therefore, increasing the bond between the steel fibers or rCF with the concrete matrix could increase the tensile capacity of the concrete significantly.

The mechanism involved with optimizing concrete mix design with carbon fibers involves lengths of chopped fiber to be long enough to provide bridging of tensile cracks in the cement composite phase and sufficient fiber volume fraction to augment the mechanical properties. For workability, the fiber volume fraction should be such that the rheology is not too far from the neat mix design. For optimal load transfer, one needs to optimize the interfacial shear strength between the carbon fiber surface and the cured cement phase using appropriate physical and chemical coating. For thermal insulation, increasing the trapped air/void phases uniformly distributed and small enough in size to minimize strength loss is targeted. For improved ductility, one needs to optimize fiber length distribution and fiber bridging without clumping in a well-dispersed state. Though, all of these features were not realized in this paper, we have provided controlling mechanisms and example mix designs to realize strong, light, and multifunctional concrete mix designs utilizing durable, lightweight, and low carbon footprint recycled carbon fibers.

## 5. Conclusions

In this study, recycled carbon fiber was evaluated for its mechanical properties (compression, tensile, and flexural strength) for high-strength, structural-light, and ultra-light concrete mix designs. The mechanical performance of the recycled carbon rCF-reinforced concrete mix designs was compared to traditional commercial steel-fiber-reinforced concrete and neat mixes. Scanning electron microscopy was used to examine the surface morphology of fibers coupled with energy dispersive X-ray spectroscopy to identify the elemental chemical composition of the recycled carbon fiber and steel fiber. Additionally, wide angle X-ray scattering was performed on the recycled carbon fibers to examine the crystalline parameters of the fibers. The concrete mix designs were evaluated non-destructively using high resolution micro X-ray computed tomography to visualize the voids spatially within the concrete tensile samples and to provide quantitative information on the porosity effects on mix design and the influence of incorporating recycled carbon fibers. Thermal conductivity was performed on the concrete mix designs to evaluate the effect of porosity, inclusion of recycled carbon fiber and steel fiber, and concrete mix design type. The results of the thermal conductivity were correlated with the porosity obtained from micro X-ray computed tomography and the compressive, tensile, and flexural strength. 

The following general conclusions can be drawn for the incorporation of the recycled-carbon-fiber-reinforced method into the three concrete mix designs considered in this study:

1. Incorporation of recycled carbon fibers into the three concrete types can increase the tensile and flexural strength of neat concrete.
2. The recycled carbon-fiber-reinforced concrete has slightly higher ductility compared to the neat concrete for tension and flexural strength but lower than the steel-fiber-reinforced concrete mix designs.
3. The recycled carbon fibers tended to increase the void content in the concrete, with the exception of the ultra-light mix where the fibers constrained the aggregates and allowed for their more uniform dispersion.
4. An increase in void volume content is the primary factor affecting the decrease in thermal conductivity for the concrete mix designs.
5. The addition of fibers increases the thermal conductivity of the concrete; however, the additional voids caused by the fibers can negate the effects.

It can be observed across the different mix designs that recycled carbon fiber improves the tensile and flexural strength of the concrete while not significantly decreasing the compression strength in most cases. It was observed that the steel fibers performed better in compression, tensile, and flexural strength across the board but had a higher density than the recycled carbon fiber and neat mixes. Furthermore, it was observed that the flow of the concrete mixes was significantly reduced with the addition of recycled carbon fiber, and the effects on flow were significantly magnified when the cement content was reduced. This result suggests that the fiber volume content in the concrete mixes should be based on the cementitious material volume content as opposed to the total mix volume. It is theorized that when the fibers are mixed into the volume, they are mixing into the cementitious portion of the mix and not the aggregate portion. It must be noted that there is a need for continued studies on the microstructural and chemical properties of fiber sizing and interfacial bonding with concrete mixes to enhance flowability and mechanical performance. Overall, recycled carbon fibers are a viable reinforcement for fiber-reinforced concrete and are particularly effective when incorporated in structural-light and ultra-light applications. 

## Figures and Tables

**Table 1:** Physical properties of fibers [111].

Fiber Type	Standard	Density (g/cm^3^)	Fiber Length (mm)	Diameter (µm)	Aspect Ratio
Steel fiber	ASTM A820 Type 1 [116]	7.8 [117]	13	200	65
Recycled carbon fiber (±SD)	-	1.81	1.5 ± 1.2	6.7 ± 0.8	224

Note: ±values are standard deviation.

**Table 2:** Summary of concrete mix designs: Note that all values are in the units of kg of material per m^3^ of total concrete mix.

Mix	Fiber Type	Manufactured Sand (kg/m^3^)	Type I Portland Cement (kg/m^3^)	Silica Fume (kg/m^3^)	Water (kg/m^3^)	HRWR (kg/m^3^)	Fibers (kg/m^3^)
High-strength	rCF	1077	851	67	275	6.9	36
	Steel	1077	851	67	275	6.9	156
	None	1131	851	67	275	6.9	0
	Fiber Type	Stalite (kg/m^3^)	Type I Portland Cement (kg/m^3^)	Silica Fume (kg/m^3^)	Water (kg/m^3^)	HRWR (kg/m^3^)	Fibers (kg/m^3^)
Structural-light	rCF	901	567	44	242	4.6	36
	Steel	901	567	44	242	4.6	156
	None	931	567	44	244	4.6	0
	Fiber Type	Perlite (kg/m^3^)	Type I Portland Cement (kg/m^3^)	Silica Fume (kg/m^3^)	Water (kg/m^3^)	HRWR (kg/m^3^)	Fibers (kg/m^3^)
Ultra-light	rCF	119	851	67	350	6.9	36
	Steel	119	851	67	350	6.9	156
	None	125	851	67	353	6.9	0

**Table 3:** Summary of mechanical testing for the concrete mix designs.

Mechanical Testing Type	ASTM Testing Standard	Number of Samples per Concrete Type (High-Strength, Structural-Light, Ultra-Light)
Compression	ASTM C109 [128]	3
Tension	ASTM C307 [129]	3
Flexural	ASTM C947 [131]	4

**Table 4:** Wide angle X-ray scattering structural properties of carbon fiber.

Fiber ID	002 Peak			100 Peak Position			Crystalline Parameters	
	2θ (^o^)	FWHM (^o^)	d-Spacing (Å)	2θ (^o^)	FWHM (^o^)	d-Spacing (Å)	L_c_ (Å)	La (Å)
rCF-1	25.67	5.07	3.47	44.11	5.48	2.05	14.9	33.8
rCF-2	24.42	4.76	3.64	42.74	5.70	2.11	14.3	35.9
T700	25.19	4.68	3.53	43.33	4.14	2.09	19.7	36.5

**Table 5:** Mechanical properties, flowability, and density values of the concrete mix designs.

Material	Fiber Type	Compression (MPa)	Tension (MPa)	Flexural (MPa)	Flow	Density (g/cm^3^)
High-strength	rCF	77.0 ± 1.2 (*n* = 3)	4.55 ± 0.31 (*n* = 3)	9.40 ± 0.82 (*n* = 4)	25.00%	2.22–2.24 (*n* = 2)
	steel	88.1 ± 0.8 (*n* = 3)	6.43 ± 0.58 (*n* = 3)	24.28 ± 1.06 (*n* = 4)	150+%	2.33–2.39 (*n* = 2)
	neat	90.7 ± 8.3 (*n* = 3)	4.52 ± 0.39 (*n* = 3)	7.61 ± 1.11 (*n* = 4)	150+%	2.34–2.35 (*n* = 2)
Structural- light	rCF	28.4 ± 0.1 (*n* = 3)	2.90 ± 0.14 (*n* = 3)	2.57 ± 0.60 (*n* = 4)	N/A *	1.51–1.54 (*n* = 2)
	steel	35.0 ± 9.6 (*n* = 3)	4.77 ± 0.45 (*n* = 3)	10.02 ± 1.46 (*n* = 4)	20.3%	1.78–1.80 (*n* = 2)
	neat	61.2 ± 2.9 (*n* = 3)	2.50 ± 0.24 (*n* = 3)	6.20 ± 0.88 (*n* = 4)	84.4%	1.66–1.74 (*n* = 2)
Ultra-light	rCF	21.7 ± 0.6 (*n* = 3)	3.21 ± 0.17 (*n* = 3)	5.62 ± 1.10 (*n* = 4)	1.60%	1.47–1.49 (*n* = 2)
	steel	41.4 ± 0.7 (*n* = 3)	5.87 ± 1.31 (*n* = 3)	16.32 ± 2.62 (*n* = 4)	150+%	1.57–1.79 (*n* = 2)
	neat	24.9 ± 5.3 (*n* = 3)	1.50 ± 0.13 (*n* = 3)	3.42 ± 0.84 (*n* = 4)	150+%	1.50–1.52 (*n* = 2)

Note: ±values are standard deviation and * represents the flow test not being valid due to the concrete crumbling during the test. Density values are range values for two measurements.

**Table 6:** Void volume fraction of high-strength, structural-light, and ultra-light concrete mix designs.

Concrete Type	Neat or Reinforcement Type	Void Volume Fraction (%)
High-strength	Neat	0.9
	Steel	1.4
	rCF	4.7
Structural-light	Neat	12.2
	Steel	14.9
	rCF	16.2
Ultra-light	Neat	37.1
	Steel	30.1
	rCF	27.7

**Table 7:** Thermal conductivity properties of high-strength, structural-light, and ultra-light concrete mix designs.

Concrete Type	Neat or Reinforcement Type	Thermal Conductivity (W/mK)
High-strength	Neat	1.666 (0.011)
	Steel	1.787 (0.004)
	rCF	1.502 (0.027)
Structural-light	Neat	0.752 (0.012)
	Steel	0.945 (0.024)
	rCF	0.551 (0.018)
Ultra-light	Neat	0.341 (0.003)
	Steel	0.515 (0.032)
	rCF	0.535 (0.010)

Note: Values in parentheses are standard deviation.

## Data Availability

Data in this study are available upon reasonable request.

## References

[1] Naganna S.R., Ibrahim H.A., Yap S.P., Tan C.G., Mo K.H., El-Shafie A. (2021). Insights into the Multifaceted Applications of Architectural Concrete: A State-of-the-Art Review. Arab. J. Sci. Eng..

[2] (2019). Building Code Requirements for Structural Concrete and Commentary.

[3] Breveglieri M., Czaderski C. (2022). Reinforced concrete slabs strengthened with externally bonded carbon fibre-reinforced polymer strips under long-term environmental exposure and sustained loading. Part 1: Outdoor experiments. Compos. Part C Open Access.

[4] Bonopera M., Liao W.-C., Perceka W. (2022). Experimental–theoretical investigation of the short-term vibration response of uncracked prestressed concrete members under long-age conditions. Structures.

[5] U.S. Geological Survey (2022). Mineral Commodity Summaries 2022.

[6] Koksal F., Gencel O., Kaya M. (2015). Combined effect of silica fume and expanded vermiculite on properties of lightweight mortars at ambient and elevated temperatures. Constr. Build. Mater..

[7] Mo K.H., Ling T.-C., Alengaram U.J., Yap S.P., Yuen C.W. (2017). Overview of supplementary cementitious materials usage in lightweight aggregate concrete. Constr. Build. Mater..

[8] Hooton R.D., Bickley J.A. (2014). Design for durability: The key to improving concrete sustainability. Constr. Build. Mater..

[9] Zhang B., Feng Y., Xie J., He J., Zhang Y., Cai C., Huang D., Li L. (2022). Effects of fibres on ultra-lightweight high strength concrete: Dynamic behaviour and microstructures. Cem. Concr. Compos..

[10] Younis A., El-Sherif H., Ebead U. (2022). Shear strength of recycled-aggregate concrete beams with glass-FRP stirrups. Compos. Part C Open Access.

[11] Sharifikolouei E., Canonico F., Salvo M., Baino F., Ferraris M. (2019). Vitrified and nonvitrified municipal solid wastes as ordinary Portland cement (OPC) and sand substitution in mortars. Int. J. Appl. Ceram. Technol..

[12] Karaki A., Mohammad M., Masad E., Khraisheh M. (2021). Theoretical and computational modeling of thermal properties of lightweight concrete. Case Stud. Therm. Eng..

[13] Chung S.-Y., Han T.-S., Kim S.-Y., Jay Kim J.-H., Youm K.S., Lim J.-H. (2016). Evaluation of effect of glass beads on thermal conductivity of insulating concrete using micro CT images and probability functions. Cem. Concr. Compos..

[14] Asadi I., Shafigh P., Abu Hassan Z.F.B., Mahyuddin N.B. (2018). Thermal conductivity of concrete—A review. J. Build. Eng..

[15] Real S., Gomes M.G., Moret Rodrigues A., Bogas J.A. (2016). Contribution of structural lightweight aggregate concrete to the reduction of thermal bridging effect in buildings. Constr. Build. Mater..

[16] Del Coz Díaz J.J., García Nieto P.J., Domínguez Hernández J., Suárez Sánchez A. (2009). Thermal design optimization of lightweight concrete blocks for internal one-way spanning slabs floors by FEM. Energy Build..

[17] Cavalline T.L., Gallegos J., Castrodale R.W., Freeman C., Liner J., Wall J. (2021). Influence of Lightweight Aggregate Concrete Materials on Building Energy Performance. Buildings.

[18] Schackow A., Effting C., Folgueras M.V., Güths S., Mendes G.A. (2014). Mechanical and thermal properties of lightweight concretes with vermiculite and EPS using air-entraining agent. Constr. Build. Mater..

[19] Glenn G.M., Klamczynski A.K., Chiou B.-S., Wood D., Orts W.J., Imam S.H. (2004). Lightweight Concrete Containing an Alkaline Resistant Starch-Based Aquagel. J. Polym. Environ..

[20] Clarke J.L. (1993). Structural Lightweight Aggregate Concrete.

[21] Lawson R. (1987). Fire resistance of ribbed concrete and composite slabs. Concrete.

[22] Thienel C., Peck M. (2007). Die Renaissance leichter Betone in der Architektur. Detail.

[23] Thienel K.-C., Haller T., Beuntner N. (2020). Lightweight Concrete—From Basics to Innovations. Materials.

[24] Akers D.J., Gruber R.D., Ramme B.W., Boyle M.J., Grygar J.G., Rowe S.K., Bremner T.W., Kluckowski E.S., Sheetz S.R., Burg R.G. (2003). Guide for Structural Lightweight-Aggregate Concrete.

[25] Yu Q.L., Spiesz P., Brouwers H.J.H. (2015). Ultra-lightweight concrete: Conceptual design and performance evaluation. Cem. Concr. Compos..

[26] Zhao S., Ding X., Zhao M., Li C., Pei S. (2017). Experimental study on tensile strength development of concrete with manufactured sand. Constr. Build. Mater..

[27] Altuki R., Tyler Ley M., Cook D., Jagan Gudimettla M., Praul M. (2022). Increasing sustainable aggregate usage in concrete by quantifying the shape and gradation of manufactured sand. Constr. Build. Mater..

[28] Kiran T., Yadav S.K., Anand N., Mathews M.E., Andrushia D., Lubloy E., Kodur V. (2022). Performance evaluation of lightweight insulating plaster for enhancing the fire endurance of high strength structural concrete. J. Build. Eng..

[29] Wu T., Yang X., Wei H., Liu X. (2019). Mechanical properties and microstructure of lightweight aggregate concrete with and without fibers. Constr. Build. Mater..

[30] Kosmatka S.H., Panarese W.C., Kerkhoff B. (2002). Design and Control of Concrete Mixtures.

[31] Lab R.H. (2007). Think Formwork—Reduce Costs. Structure.

[32] Bertolini L., Elsener B., Pedeferri P., Redaelli E., Polder R.B. (2013). Corrosion of Steel in Concrete: Prevention, Diagnosis, Repair.

[33] Shi X., Xie N., Fortune K., Gong J. (2012). Durability of steel reinforced concrete in chloride environments: An overview. Constr. Build. Mater..

[34] Samples L.M., Ramirez J.A. (1999). Methods of Corrosion Protection and Durability of Concrete Bridge Decks Reinforced with Epoxy-Coated Bars—Phase I.

[35] Yan D., Qian H., Xu Z., Chen S., Chen G. (2020). Microstructural and mechanical characterization of the interface between concrete and chemically reactive enamel (CRE) coated rebar. Constr. Build. Mater..

[36] Di Franco F., Zaffora A., Megna B., Santamaria M. (2021). Heterogeneous crystallization of zinc hydroxystannate on galvanized steel for enhancing the bond strength at the rebar/concrete interface. Chem. Eng. J..

[37] Merli R., Preziosi M., Acampora A., Lucchetti M.C., Petrucci E. (2020). Recycled fibers in reinforced concrete: A systematic literature review. J. Clean. Prod..

[38] Al-Kharabsheh B.N., Arbili M.M., Majdi A., Alogla S.M., Hakamy A., Ahmad J., Deifalla A.F. (2023). Basalt Fiber Reinforced Concrete: A Compressive Review on Durability Aspects. Materials.

[39] De Carvalho Bello C.B., Cecchi A. (2017). Experiments on natural fibers: Durability and mechanical properties. Adv. Mater. Process. Technol..

[40] Baraldi D., Boscato G., Cecchi A., de Carvalho Bello C.B. (2022). An Updated Discrete Element Model for the In-Plane Behaviour of NFRCM Strengthed Masonry Walls. Key Eng. Mater..

[41] Banthia N., Sappakittipakorn M. (2007). Toughness enhancement in steel fiber reinforced concrete through fiber hybridization. Cem. Concr. Res..

[42] Di Prisco M., Plizzari G., Vandewalle L. (2009). Fibre reinforced concrete: New design perspectives. Mater. Struct..

[43] Ghanem S.Y., Bowling J., Sun Z. (2021). Mechanical Properties of Hybrid Synthetic Fiber Reinforced Self- Consolidating Concrete. Compos. Part C Open Access.

[44] Song P.S., Wu J.C., Hwang S., Sheu B.C. (2005). Assessment of statistical variations in impact resistance of high-strength concrete and high-strength steel fiber-reinforced concrete. Cem. Concr. Res..

[45] Betterman L.R., Ouyang C., Shah S.P. (1995). Fiber-matrix interaction in microfiber-reinforced mortar. Adv. Cem. Based Mater..

[46] Brandt A.M. (2008). Fibre reinforced cement-based (FRC) composites after over 40 years of development in building and civil engineering. Compos. Struct..

[47] Rossi P. (2001). Ultra-High Performance Fiber-Reinforced Concretes. Concr. Int..

[48] Afroughsabet V., Biolzi L., Ozbakkaloglu T. (2016). High-performance fiber-reinforced concrete: A review. J. Mater. Sci..

[49] Mehta P.K., Monteiro P.J. (2014). Concrete: Microstructure, Properties, and Materials.

[50] Biolzi L., Cattaneo S., Guerrini G.L. (2000). Fracture of Plain and Fiber-Reinforced High Strength Mortar Slabs with EA and ESPI Monitoring. Appl. Compos. Mater..

[51] Zhou H., Jia B., Huang H., Mou Y. (2020). Experimental Study on Basic Mechanical Properties of Basalt Fiber Reinforced Concrete. Materials.

[52] Dvorkin L., Dvorkin O. (2006). Basics of Concrete Science.

[53] Barros J.A., Sena-Cruz J. (2001). Fracture energy of steel fiber-reinforced concrete. Mech. Compos. Mater. Struct..

[54] Mendis P. (2003). Design of high-strength concrete members: State-of-the-art. Prog. Struct. Eng. Mater..

[55] Shah S.P., Ahmad S.H. (1994). High Performance Concrete: Properties and Applications.

[56] Malier Y. (1992). High Performance Concrete: From Material to Structure.

[57] Gjørv O. (2008). High-strength concrete. Developments in the Formulation and Reinforcement of Concrete.

[58] Mendis P., Pendyala R. High-strength/High-performance Concrete in Australia-Design and Applications. Proceedings of the 4th World Conference on Utilization of High-Strength/High-Performance Concrete.

[59] Chang P.-K., Hwang C.-L., Peng Y.-N. (2001). Application of High-Performance Concrete to High-Rise Building in Taiwan. Adv. Struct. Eng..

[60] French C., Mokhtarzadeh A., Ahlborn T., Leon R. (1998). High-strength concrete applications to prestressed bridge girders. Constr. Build. Mater..

[61] Persson B.S., Johansson A.G., Johansson P.S. (1999). Prefabrication with HSC. Concr. Int..

[62] Thornton C.H., Mohamad H., Hungspruke U., Joseph L.M., Hashimah H. (1999). The Petronas Twin Towers and High-Performance Concrete. ACI Symp. Publ..

[63] Tadros M.K., Huo X., Ma Z.J., Baishya M. (2000). Structural Design of High-Performance Concrete Bridges. ACI Symp. Publ..

[64] Breitenbucher R. (1998). Developments and applications of high-performance concrete. Mater. Struct..

[65] Balaguru P., Foden A. (1996). Properties of Fiber Reinforced Structural Lightweight Concrete. ACI Struct. J..

[66] Wang J.-Y., Chia K.-S., Liew J.-Y.R., Zhang M.-H. (2013). Flexural performance of fiber-reinforced ultra lightweight cement composites with low fiber content. Cem. Concr. Compos..

[67] Ohama Y. (1989). Carbon-cement composites. Carbon.

[68] Ali M.A., Majumdar A.J., Rayment D.L. (1972). Carbon fibre reinforcement of cement. Cem. Concr. Res..

[69] Daniel I.M., Ishai O., Daniel I.M., Daniel I. (2006). Engineering Mechanics of Composite Materials.

[70] Kizilkanat A.B. (2016). Experimental Evaluation of Mechanical Properties and Fracture Behavior of Carbon Fiber Reinforced High Strength Concrete. Period. Polytech. Civ. Eng..

[71] Chen B., Liu J. (2005). Contribution of hybrid fibers on the properties of the high-strength lightweight concrete having good workability. Cem. Concr. Res..

[72] Isa M.N., Pilakoutas K., Guadagnini M., Angelakopoulos H. (2020). Mechanical performance of affordable and eco-efficient ultra-high performance concrete (UHPC) containing recycled tyre steel fibres. Constr. Build. Mater..

[73] Zhong R., Wille K., Viegas R. (2018). Material efficiency in the design of UHPC paste from a life cycle point of view. Constr. Build. Mater..

[74] Das S. (2011). Life cycle assessment of carbon fiber-reinforced polymer composites. Int. J. Life Cycle Assess..

[75] Van de Werken N., Reese M.S., Taha M.R., Tehrani M. (2019). Investigating the effects of fiber surface treatment and alignment on mechanical properties of recycled carbon fiber composites. Compos. Part A Appl. Sci. Manuf..

[76] Pimenta S., Pinho S.T. (2011). Recycling carbon fibre reinforced polymers for structural applications: Technology review and market outlook. Waste Manag..

[77] Butenegro J.A., Bahrami M., Abenojar J., Martínez M.Á. (2021). Recent Progress in Carbon Fiber Reinforced Polymers Recycling: A Review of Recycling Methods and Reuse of Carbon Fibers. Materials.

[78] Ary Subagia I.D.G., Kim Y., Tijing L.D., Kim C.S., Shon H.K. (2014). Effect of stacking sequence on the flexural properties of hybrid composites reinforced with carbon and basalt fibers. Compos. Part B Eng..

[79] Mainka H., Täger O., Körner E., Hilfert L., Busse S., Edelmann F.T., Herrmann A.S. (2015). Lignin—An alternative precursor for sustainable and cost-effective automotive carbon fiber. J. Mater. Res. Technol..

[80] Hassan M.M., Schiermeister L., Staiger M.P. (2015). Sustainable Production of Carbon Fiber: Effect of Cross-Linking in Wool Fiber on Carbon Yields and Morphologies of Derived Carbon Fiber. ACS Sustain. Chem. Eng..

[81] Ghosh T., Kim H.C., De Kleine R., Wallington T.J., Bakshi B.R. (2021). Life cycle energy and greenhouse gas emissions implications of using carbon fiber reinforced polymers in automotive components: Front subframe case study. Sustain. Mater. Technol..

[82] Danish A., Mosaberpanah M.A., Salim M.U., Amran M., Fediuk R., Ozbakkaloglu T., Rashid M.F. (2022). Utilization of recycled carbon fiber reinforced polymer in cementitious composites: A critical review. J. Build. Eng..

[83] Mohamed Sultan A.A., Mativenga P.T. (2019). Sustainable Location Identification Decision Protocol (SuLIDeP) for determining the location of recycling centres in a circular economy. J. Clean. Prod..

[84] Akbar A., Kodur V.K.R., Liew K.M. (2021). Microstructural changes and mechanical performance of cement composites reinforced with recycled carbon fibers. Cem. Concr. Compos..

[85] Howarth J., Mareddy S.S.R., Mativenga P.T. (2014). Energy intensity and environmental analysis of mechanical recycling of carbon fibre composite. J. Clean. Prod..

[86] Karuppannan Gopalraj S., Kärki T. (2020). A review on the recycling of waste carbon fibre/glass fibre-reinforced composites: Fibre recovery, properties and life-cycle analysis. SN Appl. Sci..

[87] Hadigheh S.A., Wei Y., Kashi S. (2021). Optimisation of CFRP composite recycling process based on energy consumption, kinetic behaviour and thermal degradation mechanism of recycled carbon fibre. J. Clean. Prod..

[88] Pickering S., Turner T., Meng F., Morris C., Heil J., Wong K., Melendi-Espina S. (2015). Developments in the fluidised bed process for fibre recovery from thermoset composites. Proceedings of the 2nd Annual Composites and Advanced Materials Expo, CAMX 2015.

[89] Pimenta S., Pinho S.T., Robinson P., Wong K.H., Pickering S.J. (2010). Mechanical analysis and toughening mechanisms of a multiphase recycled CFRP. Compos. Sci. Technol..

[90] Zhu J.-H., Chen P.-y., Su M.-n., Pei C., Xing F. (2019). Recycling of carbon fibre reinforced plastics by electrically driven heterogeneous catalytic degradation of epoxy resin. Green Chem..

[91] Kumar S., Krishnan S. (2020). Recycling of carbon fiber with epoxy composites by chemical recycling for future perspective: A review. Chem. Pap..

[92] Verma S., Balasubramaniam B., Gupta R.K. (2018). Recycling, reclamation and re-manufacturing of carbon fibres. Curr. Opin. Green Sustain. Chem..

[93] Bledzki A.K., Seidlitz H., Krenz J., Goracy K., Urbaniak M., Rösch J.J. (2020). Recycling of Carbon Fiber Reinforced Composite Polymers—Review—Part 2: Recovery and Application of Recycled Carbon Fibers. Polymers.

[94] Ma C., Sánchez-Rodríguez D., Kamo T. (2020). Influence of thermal treatment on the properties of carbon fiber reinforced plastics under various conditions. Polym. Degrad. Stab..

[95] Karuppannan Gopalraj S., Kärki T. (2020). A Study to Investigate the Mechanical Properties of Recycled Carbon Fibre/Glass Fibre-Reinforced Epoxy Composites Using a Novel Thermal Recycling Process. Processes.

[96] Sun H., Guo G., Memon S.A., Xu W., Zhang Q., Zhu J.-H., Xing F. (2015). Recycling of carbon fibers from carbon fiber reinforced polymer using electrochemical method. Compos. Part A Appl. Sci. Manuf..

[97] Nahil M.A., Williams P.T. (2011). Recycling of carbon fibre reinforced polymeric waste for the production of activated carbon fibres. J. Anal. Appl. Pyrolysis.

[98] Das M., Chacko R., Varughese S. (2018). An efficient method of recycling of CFRP waste using peracetic acid. ACS Sustain. Chem. Eng..

[99] Ma Y., Nutt S. (2018). Chemical treatment for recycling of amine/epoxy composites at atmospheric pressure. Polym. Degrad. Stab..

[100] Ogi K., Shinoda T., Mizui M. (2005). Strength in concrete reinforced with recycled CFRP pieces. Compos. Part A Appl. Sci. Manuf..

[101] Mastali M., Dalvand A. (2016). The impact resistance and mechanical properties of self-compacting concrete reinforced with recycled CFRP pieces. Compos. Part B Eng..

[102] Mastali M., Dalvand A., Sattarifard A. (2017). The impact resistance and mechanical properties of the reinforced self-compacting concrete incorporating recycled CFRP fiber with different lengths and dosages. Compos. Part B Eng..

[103] Akbar A., Liew K.M. (2020). Influence of elevated temperature on the microstructure and mechanical performance of cement composites reinforced with recycled carbon fibers. Compos. Part B Eng..

[104] Wang Y., Zhang S., Luo D., Shi X. (2019). Effect of chemically modified recycled carbon fiber composite on the mechanical properties of cementitious mortar. Compos. Part B Eng..

[105] Liew K.M., Pan Z., Zhang L.-W. (2019). The recent progress of functionally graded CNT reinforced composites and structures. Sci. China Phys. Mech. Astron..

[106] Meek N., Penumadu D. (2021). Nonlinear elastic response of pan based carbon fiber to tensile loading and relations to microstructure. Carbon.

[107] Voet A., Morawski J.-C., Donnet J.-B. (1975). Dynamic mechanical properties of carbon fibers. Carbon.

[108] Paulauskas F.L., White T.L., Spruiell J.E. (2006). Structure and Properties of Carbon Fibers Produced Using Microwave-Assisted Plasma Technology, Part 2.

[109] Lu H., Alymov E., Shah S., Peterson K. (2017). Measurement of air void system in lightweight concrete by X-ray computed tomography. Constr. Build. Mater..

[110] Mohammad M., Masad E., Seers T., Al-Ghamdi S.G. (2020). Properties and Microstructure Distribution of High-Performance Thermal Insulation Concrete. Materials.

[111] Patchen A., Young S., Penumadu D. (2023). An Investigation of Mechanical Properties of Recycled Carbon Fiber Reinforced Ultra-High-Performance Concrete. Materials.

[112] Han Z., Zhang Y., Zhang W., Qiao H., Feng Q., Xue C., Shang M. (2023). Study on comprehensive morphological parameters of manufactured sand based on CT scanning and entropy method and its application in rheology of manufactured sand mortar. Constr. Build. Mater..

[113] Youm K.-S., Moon J., Cho J.-Y., Kim J.J. (2016). Experimental study on strength and durability of lightweight aggregate concrete containing silica fume. Constr. Build. Mater..

[114] Liu K., Lu L., Wang F., Liang W. (2017). Theoretical and experimental study on multi-phase model of thermal conductivity for fiber reinforced concrete. Constr. Build. Mater..

[115] Cook D.J., Uher C. (1974). The thermal conductivity of fibre-reinforced concrete. Cem. Concr. Res..

[116] (2022). Standard Specification for Steel Fibers for Fiber-Reinforced Concrete.

[117] Kang S.-T., Lee Y., Park Y.-D., Kim J.-K. (2010). Tensile fracture properties of an Ultra High Performance Fiber Reinforced Concrete (UHPFRC) with steel fiber. Compos. Struct..

[118] Anderson D.P. (1991). Carbon Fiber Morphology. 2. Expanded Wide-Angle X-ray Diffraction Studies of Carbon Fibers.

[119] Akbar A., Liew K.M. (2020). Assessing recycling potential of carbon fiber reinforced plastic waste in production of eco-efficient cement-based materials. J. Clean. Prod..

[120] Toray Composite Materials America I. TORAYCA® Carbon Fiber. Toraycma.com/products/carbon-fiber/#pattern2_1.

[121] Yogendran V., Langan B.W., Haque M.N., Ward M.A. (1987). Silica Fume in High-Strength Concrete. ACI Mater. J..

[122] Brunauer S. (1962). Tobermorite gel—The heart of concrete. Am. Sci..

[123] Park S.B., Lee B.I., Lim Y.S. (1991). Experimental study on the engineering properties of carbon fiber reinforced cement composites. Cem. Concr. Res..

[124] Katz A., Li V.C., Kazmer A. (1995). Bond Properties of Carbon Fibers in Cementitious Matrix. J. Mater. Civ. Eng..

[125] (2016). Standard Test Method for Relative Density (Specific Gravity) and Absorption of Fine Aggregate.

[126] Aggerate S.L. Properties & Gradations. https://www.stalite.com/material-gradations.

[127] (2020). Standard Test Method for Flow of Hydraulic Cement Mortar.

[128] (2021). Standard Test Method for Compressive Strength of Hydraulic Cement Mortars (Using 2-in. or [50 mm] Cube Specimens).

[129] (2018). Standard Test Method for Tensile Strength of Chemical-Resistant Mortar, Grouts, and Monolithic Surfacings.

[130] Fixtures W.T. Long Beam Flexure Test Fixture (ASTM C393) Model No. WTF-LF (Aluminum and 17-4PH Stainless Steel). https://wyomingtestfixtures.com/products/flexural/long-beam-flexure-test-fixture-astm-c-393/.

[131] (2016). Standard Test Method for Flexural Properties of Thin-Section Glass-Fiber-Reinforced Concrete (Using Simple Beam with Third-Point Loading).

[132] Qsymah A., Sharma R., Yang Z., Margetts L., Mummery P. (2017). Micro X-ray computed tomography image-based two-scale homogenisation of ultra high performance fibre reinforced concrete. Constr. Build. Mater..

[133] Vicente M.A., Mínguez J., González D.C. (2019). Computed tomography scanning of the internal microstructure, crack mechanisms, and structural behavior of fiber-reinforced concrete under static and cyclic bending tests. Int. J. Fatigue.

[134] Arwood Z., Cousins D.S., Young S., Stebner A.P., Penumadu D. (2023). Infusible thermoplastic composites for wind turbine blade manufacturing: Static characterization of thermoplastic laminates under ambient conditions. Compos. Part C Open Access.

[135] Cotton R.T., Pearce C.W., Young P.G., Kota N., Leung A.C., Bagchi A., Qidwai S.M. (2016). Development of a geometrically accurate and adaptable finite element head model for impact simulation: The Naval Research Laboratory–Simpleware Head Model. Comput. Methods Biomech. Biomed. Eng..

[136] (2022). Plastics—Determination of Thermal Conductivity and Thermal Diffusivity—Part 2: Transient Plane Heat Source (Hot Disc) Method.

[137] Hot Disk AB (2016). Hot Disk Thermal Constants Analyser Instruction Manual.

[138] He Y. (2005). Rapid thermal conductivity measurement with a hot disk sensor: Part 1. Theoretical considerations. Thermochim. Acta.

[139] Rahaman M.S.A., Ismail A.F., Mustafa A. (2007). A review of heat treatment on polyacrylonitrile fiber. Polym. Degrad. Stab..

[140] Xu Y., Chung D.D.L. (2001). Silane-treated carbon fiber for reinforcing cement. Carbon.

